# Assembly and stoichiometry of the core structure of the bacterial flagellar type III export gate complex

**DOI:** 10.1371/journal.pbio.2002281

**Published:** 2017-08-03

**Authors:** Takuma Fukumura, Fumiaki Makino, Tobias Dietsche, Miki Kinoshita, Takayuki Kato, Samuel Wagner, Keiichi Namba, Katsumi Imada, Tohru Minamino

**Affiliations:** 1 Graduate School of Frontier Biosciences, Osaka University, Suita, Osaka, Japan; 2 Interfactulty Institute of Microbiology and Infection Medicine, Section of Cellular and Molecular Microbiology, Eberhard Karls University Tübingen, Tübingen, Germany; 3 German Center for Infection Research (DZIF), Partner-site Tübingen, Tübingen, Germany; 4 Quantitative Biology Center, Riken, Suita, Osaka, Japan; 5 Department of Macromolecular Science, Graduate School of Science, Osaka University, Toyonaka, Osaka, Japan; UMDNJ/Robert Wood Johnson Medical School, United States of America

## Abstract

The bacterial flagellar type III export apparatus, which is required for flagellar assembly beyond the cell membranes, consists of a transmembrane export gate complex and a cytoplasmic ATPase complex. FlhA, FlhB, FliP, FliQ, and FliR form the gate complex inside the basal body MS ring, although FliO is required for efficient export gate formation in *Salmonella enterica*. However, it remains unknown how they form the gate complex. Here we report that FliP forms a homohexameric ring with a diameter of 10 nm. Alanine substitutions of conserved Phe-137, Phe-150, and Glu-178 residues in the periplasmic domain of FliP (FliP_P_) inhibited FliP_6_ ring formation, suppressing flagellar protein export. FliO formed a 5-nm ring structure with 3 clamp-like structures that bind to the FliP_6_ ring. The crystal structure of FliP_P_ derived from *Thermotoga maritia*, and structure-based photo-crosslinking experiments revealed that Phe-150 and Ser-156 of FliP_P_ are involved in the FliP–FliP interactions and that Phe-150, Arg-152, Ser-156, and Pro-158 are responsible for the FliP–FliO interactions. Overexpression of FliP restored motility of a ∆*fliO* mutant to the wild-type level, suggesting that the FliP_6_ ring is a functional unit in the export gate complex and that FliO is not part of the final gate structure. Copurification assays revealed that FlhA, FlhB, FliQ, and FliR are associated with the FliO/FliP complex. We propose that the assembly of the export gate complex begins with FliP_6_ ring formation with the help of the FliO scaffold, followed by FliQ, FliR, and FlhB and finally FlhA during MS ring formation.

## Author summary

The bacterial flagellar type III export gate complex is a membrane-embedded nanomachine responsible for flagellar protein export and exits in a patch of membrane within the central pore of the basal body MS ring. In this work, we investigate how formation of the export gate complex is initiated. The export gate complex is composed of 5 highly conserved transmembrane proteins: FlhA, FlhB, FliP, FliQ, and FliR. Each subunit protein assembles into the gate during MS ring formation in a well-coordinated manner. The transmembrane protein FliO is required for efficient assembly of the export gate complex in *S*. *enterica* but is not essential for flagellar protein export. Here we carry out biochemical and structural analyses of FliP and provide direct evidence suggesting that FliP forms a trimer-of-dimer structure with a diameter of 10 nm. The assembly of the export gate complex begins with FliP_6_ ring formation with the help of the FliO scaffold, followed by FliQ, FliR, and FlhB and finally FlhA during MS ring formation. Given the structural and functional similarities between the flagellar and the virulence-factor-delivering injectisome machineries, we propose that the periplasmic domain of FliP homologues of the injectisome could be a good target for novel antibiotics.

## Introduction

The bacterial flagellum is supramolecular motility machinery consisting of basal body rings and an axial structure consisting of the rod, the hook, the hook-filament junction, the filament, and the filament cap. Flagellar axial proteins are translocated across the cytoplasmic membrane by a type III protein export apparatus and assemble at the distal end of the growing structure. The export apparatus consists of an export gate complex formed by 5 highly conserved transmembrane proteins (FlhA, FlhB, FliP, FliQ, and FliR) and a cytoplasmic ATPase complex consisting of FliH, FliI, and FliJ [1–4]. These flagellar proteins are evolutionarily related to the components of the type III secretion system (T3SS) of pathogenic bacteria, also known as the injectisome [5]. The transmembrane protein, FliO, which is not conserved in flagellar and virulence-associated T3SS family, is required for efficient assembly of the export gate complex in *S*. *enterica* (hereafter referred to as *Salmonella*) but is not essential for flagellar protein export [6–8].

The flagellar type III export apparatus utilizes ATP and proton motive force across the cytoplasmic membrane to drive protein export [2,3]. Recently, it has been shown that ATP hydrolysis by the FliI ATPase and the following rapid protein translocation by the export gate complex are both linked to efficient proton translocation through the gate, suggesting that the export apparatus acts as a proton/protein antiporter to couple the proton flow through the gate with protein export [9]. Interestingly, the structure of the cytoplasmic ATPase complex looks similar to those of F- and V-type rotary ATPases [10–12].

The export gate complex is located inside the basal body MS ring formed by a transmembrane protein, FliF [13,14]. FlhA forms a homononamer [8,13] and acts as an energy transducer along with the cytoplasmic ATPase complex [15–19]. The C-terminal cytoplasmic domains of FlhA and FlhB form a docking platform for the ATPase complex, flagellar type III export chaperones, and export substrates [20–22] and coordinate flagellar protein export with assembly [23–26]. Genetic analyses have suggested possible interactions of the N-terminal transmembrane domain of FlhA (FlhA_TM_) with FliF [27], FliR [28] and FlhB [29]. Since a FlhB–FliR fusion protein is partially functional in *Salmonella*, FlhB presumably associates with FliR in a 1-to-1 fashion [30]. FliP and FliR are incorporated into the basal body at the earliest stage of MS ring formation [31,32]. The transmembrane export gate complex of the *Salmonella* SPI-1 T3SS is composed of SpaP (FliP homologue), SpaQ (FliQ homologue), SpaR (FliR homologue), SpaS (FlhB homologue), and InvA (FlhA homologue) in a 5:1:1:1:9 stoichiometry [33]. Recently, it has been shown that 5 copies of SpaP and 1 copy of SpaR form a donut-shaped structure with a diameter of about 8 nm [34]. Since the assembly of the export apparatus begins with SpaP, SpaQ, and SpaR, followed by the assembly of SpaS and finally of InvA in the *Salmonella* SPI-1 T3SS [34,35], the assembly of the flagellar export gate complex is postulated to occur in a way similar to the *Salmonella* SPI-1 T3SS [8].

FliP is a 25-kDa transmembrane protein that has a cleavable N-terminal signal peptide, 4 transmembrane (TM) helices, and a relatively large periplasmic domain (FliP_P_) between TM-2 and TM-3 (S1 Fig) [36]. The number of FliP molecules has been estimated to be 4 to 5 per basal body in *Salmonella* [32]. FliP_P_ of *T*. *maritia* (*Tm*-FliP_P_) forms a homotetramer in solution [37], raising the possibility that *Salmonella* FliP (*St-*FliP) forms an oligomer through interactions between FliP_P_ domains. To study the oligomeric structure of FliP, we purified *St-*FliP from the membrane fraction by solubilizing it with 1% n-dodecyl β-D-maltoside (DDM) and analyzed it by electron microscopy (EM) and image analysis. We show that FliP forms a homohexameric ring with a diameter of about 10 nm. We also determined the structure of *Tm*-FliP_P_ at 2.4 Å resolution and carried out structure-based photo-crosslinking experiments. We will discuss the assembly mechanism of the transmembrane export gate complex.

## Results

### Oligomeric state of full-length *St-*FliP

**Fig 1:**
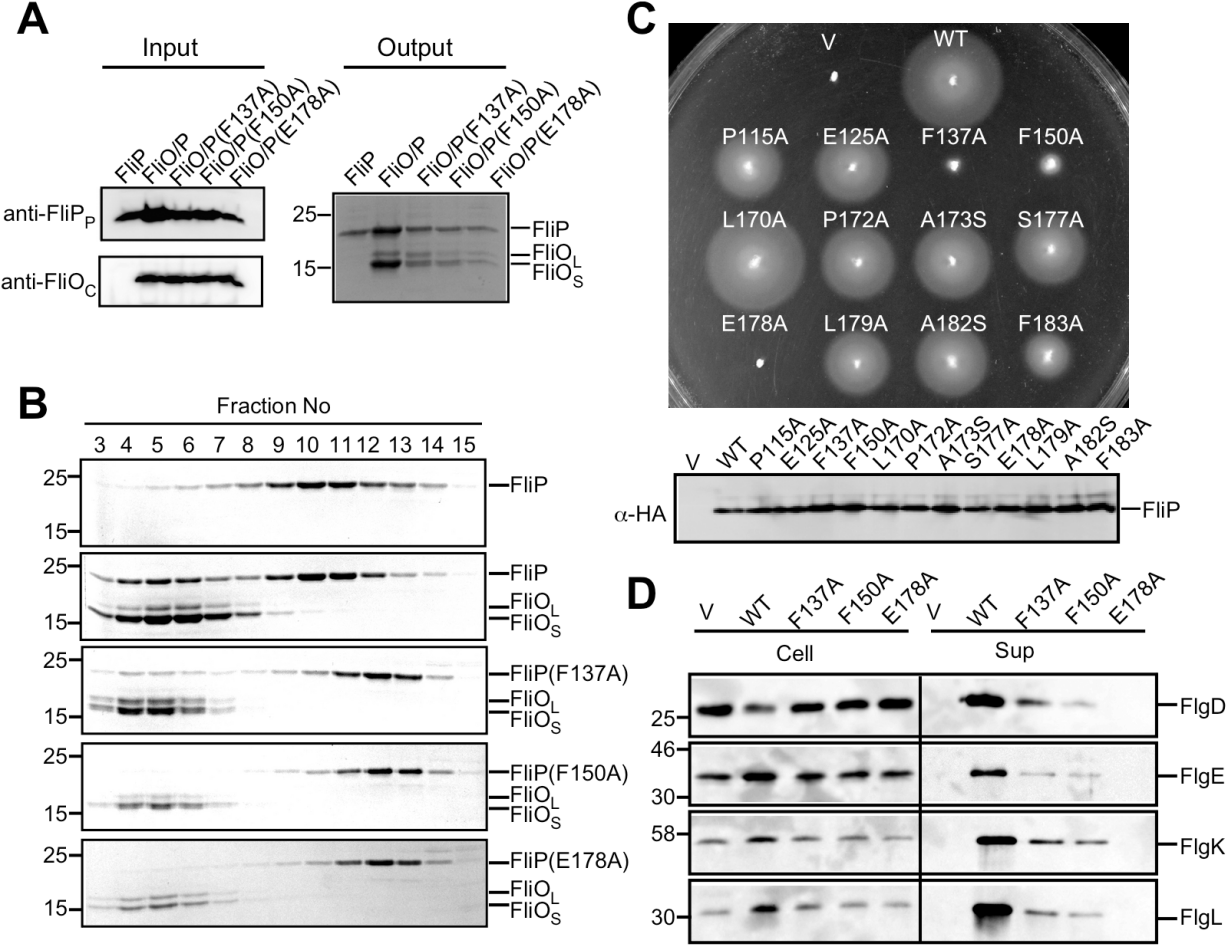
Genetic and biochemical characterization of *St-*FliP. (A) Ni affinity chromatography. The membrane fractions were prepared from SJW1368 expressing His-FliP (FliP), FliO and His-FliP (FliO/P), FliO and His-FliP(F137A) [FliO/P(F137A)], FliO and His-FliP(F150A) [FliO/P(F150A)], or FliO and His-FliP(E178A) [FliO/P(E178A)], solubilized by 1% n-dodecyl β-D-maltoside (DDM) and analyzed by immunoblotting with polyclonal anti-FliP_P_ or anti-FliO_C_ antibody (Input). The solubilized membranes were loaded onto a Ni–nitrilotriacetic acid (NTA) column. After washing the column extensively, proteins were eluted with a 50–400 mM imidazole gradient. Fractions containing His-FliP with or without FliO were analyzed by SDS-PAGE with Coomassie Brilliant Blue (CBB) staining (Output). Molecular mass markers (kDa) are shown on the left. (B) Size exclusion chromatography (SEC) analysis with a Superdex 200 10/300 column. Eluted factions were monitored by SDS-PAGE with CBB staining. (C) Motility of a *Salmonella* ∆*fliP* mutant transformed with pBAD24-based plasmids encoding wild-type FliP and its mutant variants in soft agar at 30°C for 6 h (upper panel). Immunoblotting, using monoclonal anti-HA antibody, of whole cell lysates prepared from the ∆*fliP* mutant transformed with pUC19-based plasmids encoding wild-type FliP and its mutant variants. (D) Effect of the F137A, F150A, and E178A mutations on flagellar protein export. Whole cell proteins (Cell) and culture supernatant fractions (Sup) were prepared from the ∆*fliP* mutant transformed with pBAD24 (indicated as V), pKY041 (indicated as WT), pKY041(F137A) (indicated as F137A), pKY041(F150A) (indicated as F150A), or pKY014(E178A) (indicated as E178A) and then analyzed by immunoblotting with polyclonal anti-FlgD (first row), anti-FlgE (second row), anti-FlgK (third row), or anti-FlgL (fourth row) antibody. The positions of molecular mass markers are indicated on the left.

To study the oligomeric state of mature form of FliP, we expressed, solubilized, and purified *St*-FliP. A hexahistidine tag (LHHHHHH) was inserted between Gln-22 and Leu-23 of *St-*FliP (His-*St*-FliP) for rapid and efficient purification (S1 Fig). The membrane fraction of *Salmonella* cells expressing His-*St*-FliP was solubilized by 1% DDM, and His-*St*-FliP was purified by Ni affinity chromatography (Fig 1A), followed by size exclusion chromatography (SEC) with a Superdex 200 10/300 column (Fig 1B, first row). The SEC elution profile of His-*St*-FliP showed 2 distinct peaks (S2A Fig). Many ring-shaped structures were observed by EM of negatively stained particles in the earlier peak fraction (S2B Fig, Peak 2) but not in the later one (S2B Fig, Peak 4).

**Fig 2:**
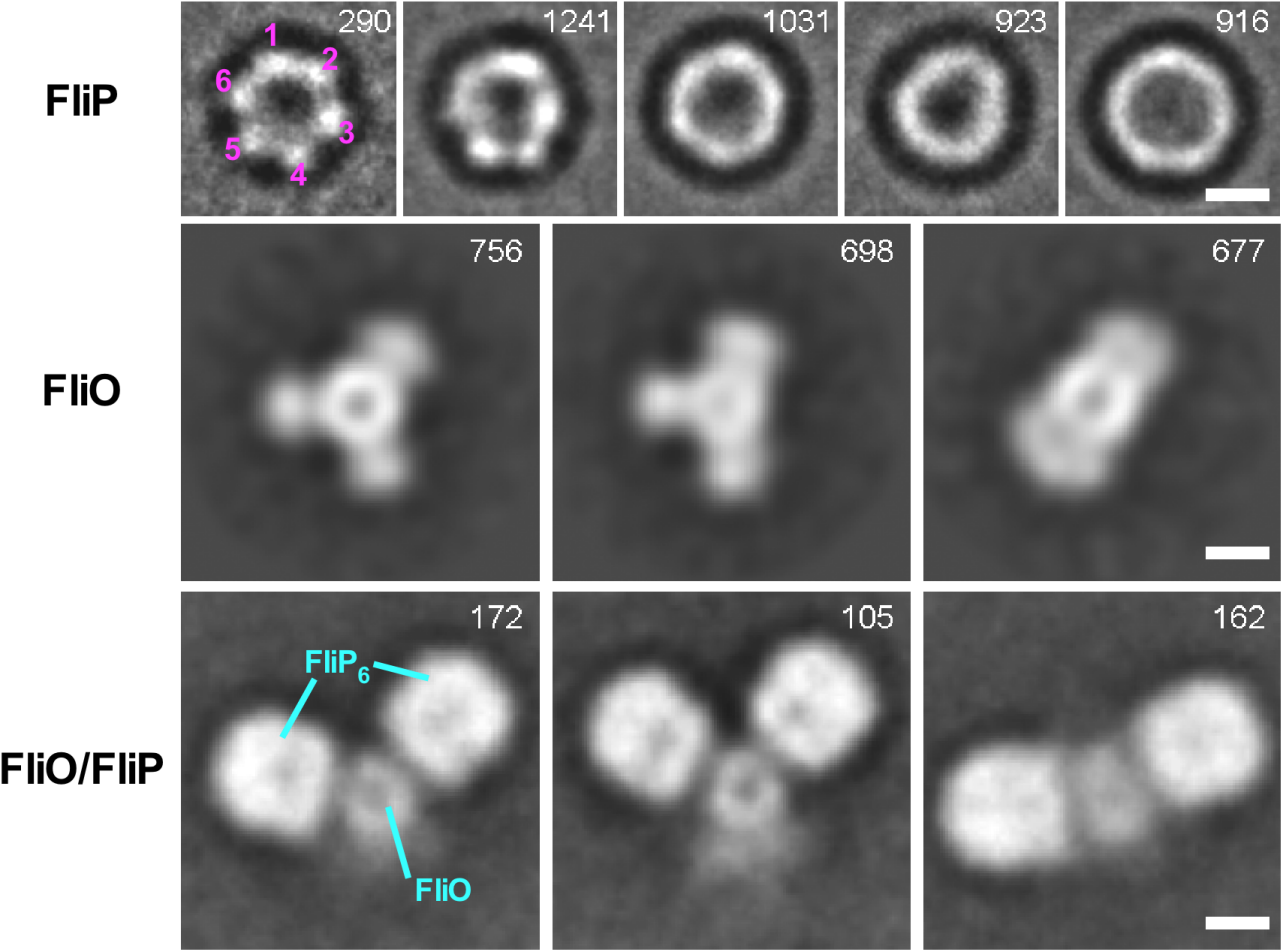
Enlarged views of representative 2D class averages of the FliP_6_ ring, the FliO complex, and the FliO/FliP complex. Reference-free 2D class average images were calculated by e2refine2d.py. All scale bars show 50 Å. The number of particles for each class is indicated in the top-right corner. FliP forms a ring structure with a diameter of about 10 nm (first row). The FliP ring has the 6-fold rotational symmetry as judged by autocorrelation analysis (see S3A, S3B and S3C Fig). FliO forms a ring structure of 5 nm in diameter with 3 flexible clamp-like structures (second row) that bind to the FliP ring with their ring axes perpendicular to the axis of the FliO ring (third row). Thus, the FliP rings in the first row are end-on views, and those in the third row are side views.

An apparent molecular mass of the FliP ring structure was estimated to be about 200 kDa by SEC (S2A Fig). Since the deduced molecular weight of His-*St*-FliP is approximately 25 kDa, the FliP ring structure presumably contains several copies of FliP together with a DDM micelle covering the transmembrane helices of FliP. To estimate the stoichiometry of the FliP ring more precisely, we carried out 2D classification EM image analysis of negatively stained FliP ring particles, followed by autocorrelation analysis for the rotational symmetry (Fig 2 and S3 Fig). The *St-*FliP rings exhibiting clear blob features were mostly hexameric with a diameter of about 10 nm (Fig 2 and S3A, S3B and S3C Fig). Autocorrelation analysis also showed that 5,333 of the 11,736 FliP ring particles analyzed were assigned to the 6-fold rotational symmetry and the rest, which did not show proper ring-shaped structures, were assigned to 5-fold or other rotational symmetries (S3D and S3E Fig), suggesting either that the ring structure is flexible or that they could be side views or incomplete partial rings.

### Interaction between FliP and FliO

Previous genetic analyses of a *Salmonella* ∆*fliO* mutant have suggested possible interactions between FliO and FliP [6,7]. To clarify this, we coexpressed FliO with His-FliP and purified them by Ni affinity chromatography and finally by SEC. In agreement with a previous report [6], FliO was expressed as 2 forms: FliO_L_ and FliO_S_ (Fig 1A). Both forms copurified with His-FliP from a SEC column (Fig 1B, second row). EM observation of the FliO/FliP complex revealed that 2 to 3 FliP_6_ rings are connected to each other through an interaction between FliP and FliO (Fig 2 and S2B and S4 Figs). Since the inner diameter of the M ring of the flagellar basal body is about 20 nm [3] and is too small to accommodate such multiring complexes of FliO and FliP, it is likely that only 1 FliP_6_ ring exists in the final structure of the export gate complex. To test this, we investigated whether overexpression of *St-*FliP restores motility of a *Salmonella* ∆*fliO* mutant. To monitor the expression level of *St-*FliP, we inserted a HA tag between Gln-22 and Leu-23 of *St-*FliP (HA-*St*-FliP). The motility of the ∆*fliO* mutant overexpressing HA-*St*-FliP was essentially the same as that of the ∆*fliO* mutant transformed with a pTrc99A-based plasmid encoding FliO (S5 Fig). This indicates that FliO is not essential for flagellar protein export.

We next tested whether FliO itself forms an oligomer. We found that the FliP_6_ ring dissociates from FliO during storage of the purified FliO/FliP complexes at 4°C (S6A Fig). Thus, we ran purified FliO/His-FliP complex samples on a Ni– nitrilotriacetic acid (NTA) column to remove His-FliP_6_ rings and the FliO/His-FliP complex, followed by SEC to purify FliO (S6B Fig). EM observation and image analysis showed that FliO forms a 5-nm ring structure with 3 flexible clamp-like structures (Fig 2 and S2 and S4 Figs). These observations led us to conclude that the FliO ring complex is not incorporated into the MS ring.

### Mutational analysis of *St*-FliP_P_

The FliP(R143H) mutation, which is located in *St-*FliP_P_, can bypass the FliO defect to some extent [6,7], raising the possibility that *St-*FliP_P_ is required for FliP_6_ ring formation. To test this, we selected relatively well-conserved residues of FliP, Pro-115, Glu-125, Phe-137, Phe-150, Leu-170, Phe-172, Ala-173, Ser-177, Glu-178, Leu-179, Ala-182, and Phe-183 (S1B Fig); replaced each residue with alanine, except for Ala-173 and Ala-182, which we replaced with serine; and then analyzed the motility of mutant strains in soft agar (Fig 1C, upper panel). These substitutions did not significantly affect the steady cellular level of FliP as judged by immunoblotting with monoclonal HA-tag antibody (Fig 1C, lower panel). HA-*St*-FliP fully restored the motility of a ∆*fliP* mutant. The L170A mutant variant complemented the ∆*fliP* mutant to the wild-type level. The P115A, E125A, P172A, A173S, S177A, L179A, A182S, and F183A mutant variants restored the motility to a considerable degree, although not to the wild-type level. The F137A and F150A mutant variants complemented the ∆*fliP* mutant to some degree, and the E178A mutant variant did not at all. In agreement with these results, the F137A and F150A mutations in FliP significantly reduced the secretion levels of the hook-capping protein FlgD, the hook protein FlgE, and the hook-filament junction proteins FlgK and FlgL, and the E178A substitution inhibited the export of these flagellar proteins (Fig 1D). These results indicate that highly conserved Phe-137, Phe-150, and Glu-178 residues of FliP_P_ are critical for the protein export activity.

To investigate whether the F137A, F150A, and E178A mutations affect the FliP–FliO interaction, we carried out copurification assays by Ni-NTA affinity chromatography. FliO coeluted with His-FliP(F137A), His-FliP(F150A), and His-FliP(E178A) from a Ni-NTA column (Fig 1A, Output), indicating that they retain the ability to bind to FliO. To test whether these FliP mutations inhibit FliP_6_ ring formation, we ran FliO/His-FliP(F137A), FliO/His-FliP(F1507A), and FliO/His-FliP(E178A) complexes on a SEC column and then analyzed the pooled fractions by EM. His-FliP(F137A), His-FliP(F150A), and His-FliP(E178A) dissociated from the FliO complex during SEC and eluted at the same position as peak 4 of wild-type FliP (Fig 1B and S2A Fig), indicating that these mutations reduced the binding affinity of FliP for FliO. The FliO ring structures were seen in their peak 3 fractions, but neither FliP(F137A), FliP(F150A), nor FliP(E178A) formed the homohexamer ring (S2B Fig, Peak 4). These results suggest that Phe-137, Phe-150, and Glu-178 in FliP_P_ contribute to the FliP–FliP interactions in the 6-fold rotational symmetry ring as well as the FliO–FliP interaction.

### Crystal structure of *Tm-*FliP_P_

**Fig 3:**
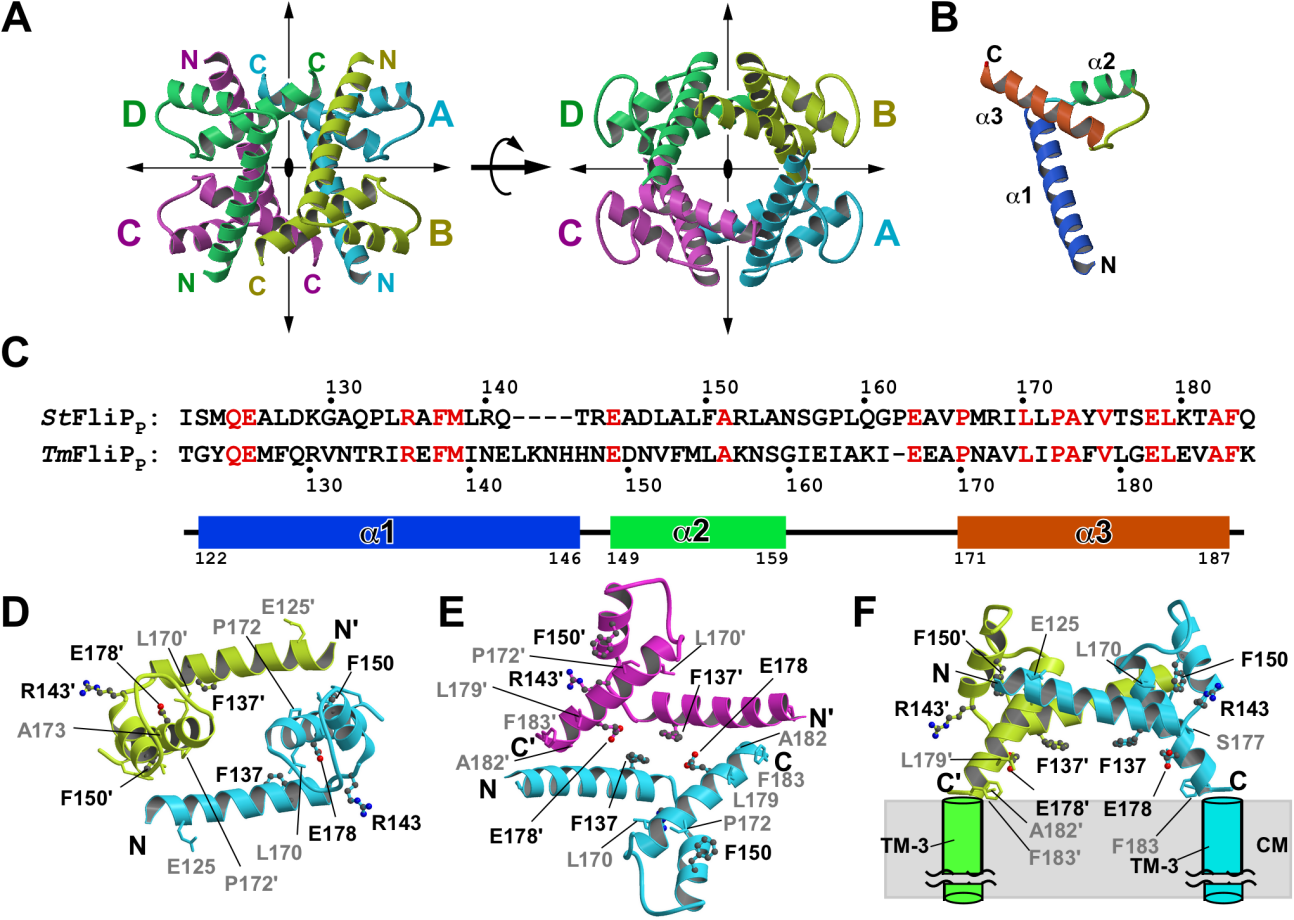
Structure of FliP_P_. (A) Ribbon diagram of the *Tm*-FliP_P_ tetramer in the crystal (Protein Data Bank [PDB] ID: 5H72). Two different views are shown. Mol A, Mol B, Mol C, and Mol D are colored in cyan, yellow green, magenta, and green, respectively. Each subunit of the *Tm-*FliP_P_ tetramer is related by D2 symmetry. (B) Cα ribbon drawing of the *Tm*-FliP_P_ monomer. The secondary structure elements are labeled with α for α-helix. (C) Structure-based sequence alignment of *Salmonella* FliP_P_ (*St*FliP_P_) and *Tm-*FliP_P_. The secondary structure of *Tm-*FliP_P_ is shown below the sequence. Identical residues are highlighted in red. Uniprot accession numbers: *Salmonella* (P54700) and *Thermotoga* (Q9WZG2). (D) Homology model of the A–B dimer of *St-*FliP_P_. (E) Homology model of the A–C dimer of *St-*FliP_P_. (F) The model of the A–B dimer connected to the TM-3 helices. Both C-termini of the A–B dimer can be directly connected to the TM-3 helices. CM, cytoplasmic membrane. Residues selected for mutational analyses are mapped and labeled in (D), (E), and (F). The residues whose substitution affected the FliP function are shown in ball-and-stick with black labels, and those that did not are in stick with gray labels.

To clarify the role of FliP_P_ in FliP_6_ ring formation, we determined the crystal structure of FliP_P_. Although no *St-*FliP_P_ crystal was obtained, the *Tm-*FliP_P_ crystals were grown [37], and its structure was solved at 2.4 Å resolution. *Tm-*FliP_P_ formed a homotetramer in the crystal (Mol A, Mol B, Mol C, and Mol D) related by pseudo D2 symmetry (Protein Data Bank [PDB] ID: 5H72) (Fig 3A). There are 2 tetramers in the asymmetric unit, and their structures are essentially identical. The 8 *Tm*-FliP_P_ molecules in the asymmetric unit show no significant structural difference (root mean square distances for Cα atoms are less than 0.46 Å for the 8 molecules). *Tm-*FliP_P_ monomer consists of 3 α-helices: α1, α2 and α3 (Fig 3B). The N-terminal 13 residues are invisible in the electron density map presumably because of their conformational flexibility. Therefore, the atomic model of *Tm*-FliP_P_ contains residues from Thr-122 to Lys-188. Since each subunit of the *Tm-*FliP_P_ tetramer is related by D2 symmetry, we studied 3 possible intermolecular interactions: between Mol A and Mol B (Mol C and Mol D), between Mol A and Mol C (Mol B and Mol D), and between Mol A and Mol D (Mol B and Mol C) (Fig 3A). The A–B interaction is hydrophobic, and Tyr-124, Phe-128, Met-154, Leu-155, Pro-176, and Leu-180 are involved in this interaction (S7A, S7C, S7D and S7E Fig). The A–C interaction contains both hydrophilic and hydrophobic nature, and Met-127, Arg-130, Val-131, Arg-134, Phe-138, Glu-142, Glu-182, Val-185, Ala-186, and Phe-187 are responsible (S7B, S7F, S7G and S7H Fig). Arg-134 forms a salt bridge with Glu-142 and Glu-182. Ala-186 and Phe-187 make hydrophobic interactions with Met-127, Val-131, and the side chain arm of Arg-130. There is no direct contact between Mol A and Mol D. Since sedimentation equilibrium analytical ultracentrifugation measurements have revealed that *Tm-*FliP_P_ forms a homotetramer in solution [37], we conclude that the tetramer structure observed in the crystal appears to be equivalent to that in solution.

### Physical contacts between *St-*FliP_P_ monomers

Although the *Tm-*FliP_P_ tetramer in the crystal is inconsistent with the *St-*FliP_6_ ring structure, it is possible that the dimer units seen in the tetramer are responsible for the hexameric ring formation of *St-*FliP if the hexamer is a trimer-of-dimer structure. Two distinct dimers are present in the *Tm-*FliP_P_ crystal: A–B dimer and A–C dimer (Fig 3A and S7 Fig). Although the sequence identity between *Tm-*FliP_P_ and *St-*FliP_P_ is only about 30% (Fig 3C), we constructed a homology model of *St-*FliP_P_ based on the *Tm-*FliP_P_ tetramer structure (Fig 3D and 3E). The interface residues are not well conserved, but the properties of the interface of *St-*FliP_P_ are similar to those of *Tm-*FliP_P_ (S8 Fig). The A–B interface of *St-*FliP_P_ is hydrophobic, and Met-123, Leu-127, Leu-149, Phe-150, and Pro-172 form the hydrophobic surface (S8A, S8B and S8C Fig). The A–C interface of *St-*FliP_P_ shows an elongated shape with both hydrophilic and hydrophobic properties (S8D, S8E and S8F Fig). Arg-140 and Gln-141 form a hydrogen-bonding network with those in the other molecule. Phe-137 is in contact with Tyr-174 and the side chain arm of Glu-178. Considering the hydrophobic nature of the A–B interface, the A–B dimer is more likely to be a dimer unit of the FliP_6_ ring structure, although the area of the A–B interface is smaller than that of the A–C interface. The C-termini of the A–B dimer can be connected to the periplasmic end of TM-3 without any steric hindrances with the cytoplasmic membrane, suggesting that the A–B dimer contributes to trimer-of-dimer ring formation (Fig 3F). Because the N-terminal 13 residues are invisible, it is possible that both of the 2 N-termini of the dimer can connect to the periplasmic ends of TM-2 helices. In contrast, if the C-termini of the A–C dimer are directly connected to TM-3, the hydrophilic surface of the dimer core region would be buried in the cytoplasmic membrane (S7H and S8F Figs), which is unlikely.

### In vivo photo-crosslinking

**Fig 4:**
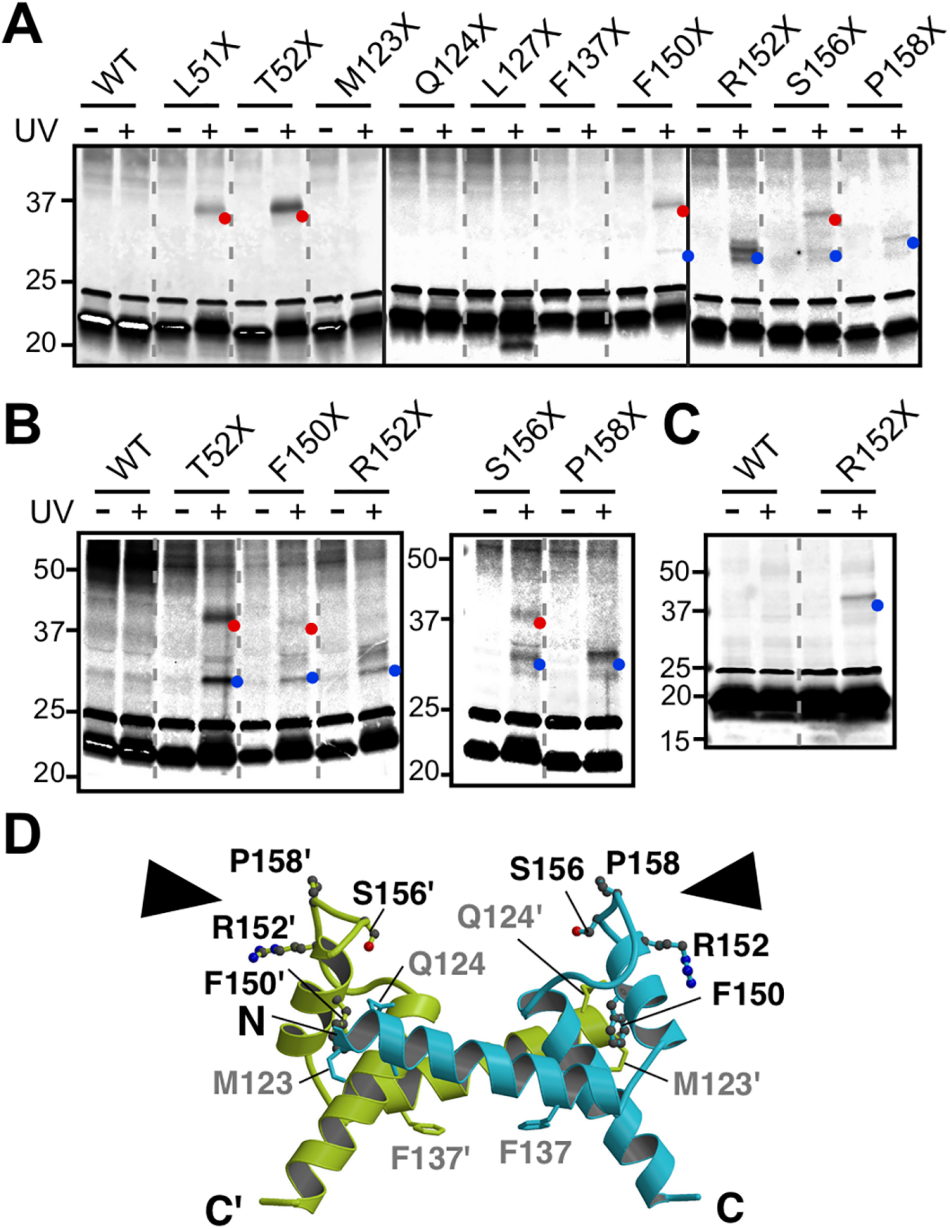
In vivo photo-crosslinking. *E*. *coli* BL21 (DE3) cells coexpressing (A) FliP-FLAG with an amber mutation at indicated positions with FliO, FliQ, and FliR, (B) FliP-FLAG with an amber mutation with FliO, or (C) FliP-HA with an amber mutation with FliO-FLAG were grown in the presence of *p*-benzoyl-phenylalanine (pBPA) and then treated with (+) or without (−) UV irradiation. Wild-type FliP-FLAG (WT) was used as a negative control. Crude membrane fractions were prepared, followed by SDS-PAGE and finally immunoblotting with monoclonal anti-FLAG antibody. Red and blue dots indicate FliP-FliP and FliP-FliO photo-crosslinked products, respectively. Each cropped blot is shown within a box. (D) The residues selected for the photo-crosslinking experiment are mapped on the A–B dimer model of *St-*FliP_P_. The residues that formed crosslinking products by the substitution with pBPA are shown in ball-and-stick with black labels, and those that did not are in stick with gray labels. Black arrowheads indicate possible interaction sites of *St-*FliP_P_ with FliO.

To investigate which dimer form is actually present in the FliP_6_ ring structure, we carried out structure-based photo-crosslinking experiments. We introduced an amber mutation at positions of 123, 124, 127, 137, 150, 152, 156, or 158 of *St-*FliP to incorporate *p*-benzoyl-phenylalanine (pBPA), which is a photoreactive phenylalanine. Since Ser-14 and Thr-15 in TM-1 of SpaP—which correspond to Leu-51 and Thr-52 in TM-1 of *St-*FliP, respectively—provide strong SpaP-SpaP photo-crosslinked products [34], we also introduced an amber mutation at positions of 51 or 52. We introduced 2 plasmids into the *Escherichia coli* BL21(DE3) strain, 1 encoding FliO, FLAG-tagged FliP (FliP-FLAG) with an amber mutation, FliQ, and FliR and the other encoding the amber suppressor tyrosyl tRNA and the engineered tyrosyl-tRNA synthetase to incorporate pBPA at the positions of amber codons. We used wild-type FliP-FLAG as a negative control. As expected, UV irradiation of pBPA at positions of 51 or 52 led to the formation of a photo-crosslinked FliP homodimer (Fig 4A, indicated by a red dot), indicating that TM-1 of FliP is responsible for the FliP–FliP interaction in a way similar to the SpaP–SpaP interaction. Both FliP(F150pBPA)-FLAG and FliP(S156pBPA)-FLAG also reproducibly gave a photo-crosslinked FliP homodimer, whereas the others did not (Fig 4A, indicated by a red dot). This photo-crosslinked product was also observed when only FliO and FliP-FLAG with an amber mutation were expressed in the presence of pBPA (Fig 4B). These results indicate that both FliP-TM1 and FliP_P_ are involved in the FliP–FliP interactions in the hexameric ring structure. Phe-150 and Ser-156 are located at the A–B interface, whereas Phe-137 is located at the A–C interface (Fig 3D and 3E and S8B and S8E Fig). Since we found that both Phe-137 and Phe-150 are required for FliP_6_ ring formation, protein export, and motility (Fig 1), the A–B dimer unit seen in the *Tm-*FliP_P_ crystal structure is likely to exist in the *St-*FliP_6_ ring structure, and it is likely that Phe-137 contributes to its trimer-of-dimer formation.

UV irradiating pBPA at positions of Phe-150, Arg-152, Ser-156, or Pro-158 produced a 30-kDa crosslinked band, suggesting the presence of a FliP-FliO crosslinked product (indicated by blue dot). This band was also present when only FliO and FliP were expressed (Fig 4B). To confirm the FliO–FliP interaction, we labeled FliO and FliP(R152pBPA) with a 3 x FLAG tag and a 3 x HA tag, respectively. FliO-FLAG formed a crosslinked band with FliP(R152pBPA)-HA but not with wild-type FliP-HA, proving the presence of the FliO–FliP_P_ interaction (Fig 4C). These results indicate that Phe-150, Arg-152, Ser-156, and Pro-158 of FliP_P_ are in relatively close proximity to FliO (Fig 4D). This is in agreement with our finding that the F150A mutation reduced the binding affinity of FliP for FliO (Fig 1). Interestingly, the UV irradiation of pBPA at a position of 52 also produced a 30-KDa crosslinked product in the absence of FliQ and FliR (Fig 4B) but not in their presence (Fig 4A), indicating that the TM-1 helix of FliP is in close proximity to FliO when FliQ and FliR are absent.

The intensity of the FliO-FliP crosslinked band formed by the introduction of pBPA at positions of Phe-150, Ser-156, or Pro-158 was weaker in the presence of FliQ and FliR (Fig 4A) than in their absence (Fig 4B), whereas the intensity of the FliP-FliP crosslinked band at positions of Phe-150 or Ser-156 was somehow stronger in the presence of FliQ and FliR (Fig 4A) than in their absence (Fig 4B). Therefore, we suggest that FliO appears to facilitate oligomerization of FliP and maintain its stability until FliQ and FliR assemble into the FliP_6_ ring and that the binding of FliQ and FliR to FliP probably induces conformational rearrangements of the FliP ring in the FliO complex.

### Interaction of the FliP_6_ ring with FliQ, FliR, FlhB, and FlhA

**Fig 5:**
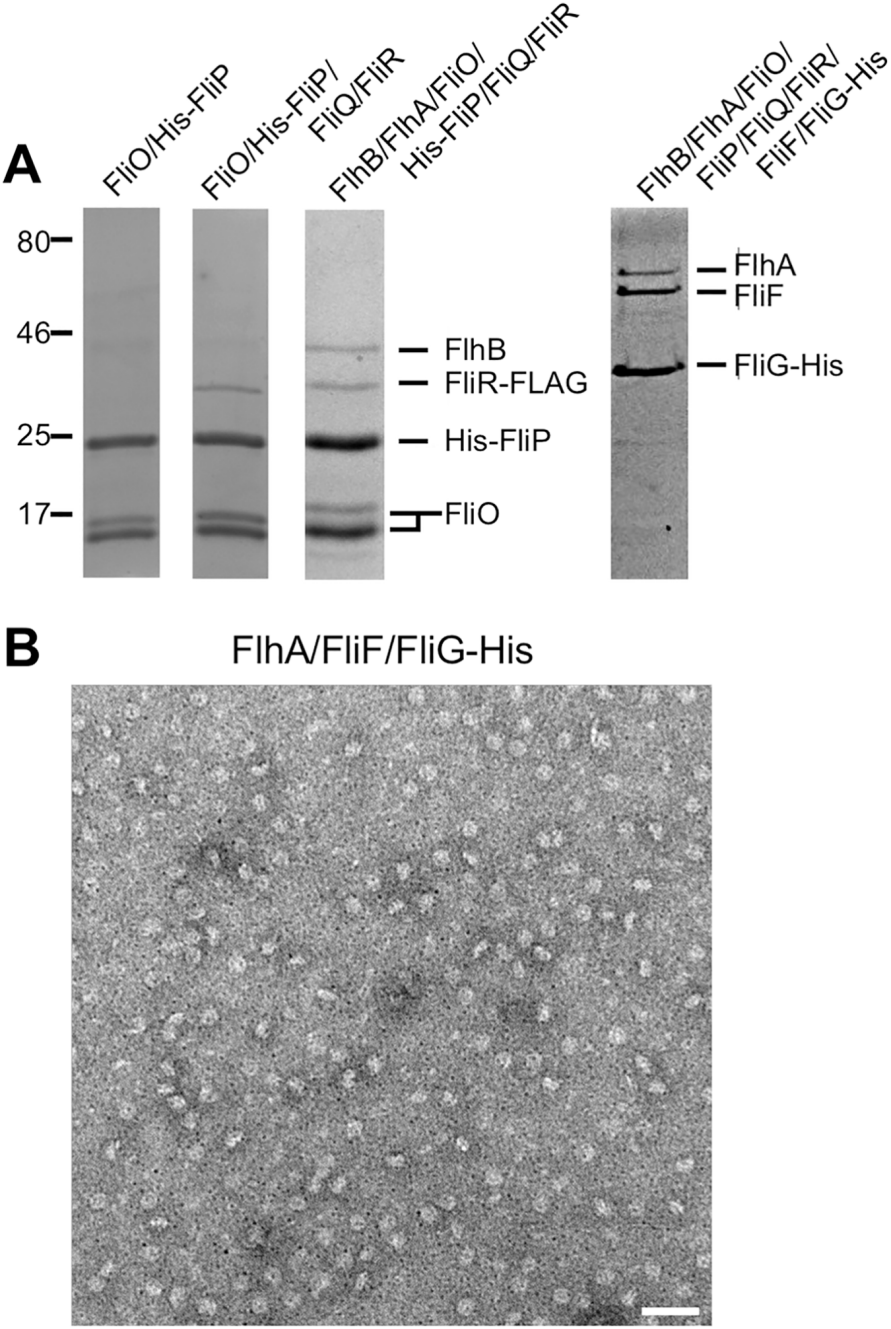
Interactions of the FliP_6_ ring with other export gate proteins. (A) SDS-PAGE of pooled fractions after size exclusion chromatography (SEC) with a Superdex 200 10/300 column. Membrane fractions were prepared from SJW1368 expressing FliO and His-FliP (lane 1); FliO, His-FliP, HA-FliQ, and FliR-FLAG (lane 2); FlhA, FlhB, FliO, His-FliP, HA-FliQ, and FliR-FLAG (lane 3); or FlhA, FlhB, FliF, FliG-His, FliO, FliP, HA-FliQ, and FliR-FLAG (lane 4) and solubilized by 1% n-dodecyl β-D-maltoside (DDM), followed by Ni affinity chromatography. For purification of the FliO/His-FliP/FliR-FLAG and FliO/His-FliP/FliR-FLAG/FlhB complexes, pooled fractions were subjected to FLAG affinity chromatography (see S9 Fig), followed by SEC with a Superdex 200 10/300 column (lanes 2 and 3). For purification of the FlhA/FliF/FliG-His complex, pooled fractions were subjected to SEC (lane 4). (B) Representative negatively stained electron microscopy (EM) images of purified FlhA/FliF/FliG-His complexes. Scale bar shows 100 nm.

To analyze the interactions of the FliP_6_ ring with other export gate proteins, we constructed plasmids coexpressing His-FliP with FliR-FLAG, with HA-FliQ and FliR-FLAG, with FliO and FliR-FLAG, with FliO, HA-FliQ, and FliR-FLAG, or with FlhA, FlhB, FliO, HA-FliQ, and FliR-FLAG. These tags did not affect the export function of export gate proteins considerably. To simplify the examination of their interactions, we expressed these proteins from a single pTrc99A-based plasmid in the *Salmonella* SJW1368 strain, in which no flagellar genes are expressed because of loss of the master regulator complex, FlhD_4_FlhC_2_ [1]. The membrane fractions of *Salmonella* cells coexpressing His-FliP with other export gate proteins were solubilized by 1% DDM, and then the proteins were purified by Ni affinity chromatography, followed by FLAG affinity chromatography (S9 Fig) and finally SEC (Fig 5A). FliR-FLAG and FlhB copurified with His-FliP and FliO, whereas neither HA-FliQ nor FlhA did (Fig 5A and S9A Fig).

It has been shown that SpaR of the *Salmonella* SPI-1 T3SS directly binds to the SpaP_5_ ring [34]. Therefore, we investigated if FliR directly binds to FliP. When only FliR-FLAG was coexpressed with His-FliP, FliR coeluted with the FliP_6_ ring from a SEC column (S9B Fig), indicating that FliR tightly associates with the FliP_6_ ring structure.

We found that FliQ and FlhA were easily dissociated from the FlhB/FliO/FliP/FliR complex. Therefore, we investigated whether the MS ring, which is made of 26 copies of a single transmembrane protein FliF, stabilizes the structure of the entire export gate complex. Since a C ring protein FliG is required for efficient MS ring formation in the cytoplasmic membrane [8], we attached a His tag to the C-terminus of FliG for efficient and rapid purification of the MS ring. To carry out copurification assay, we constructed a pTrc99A-based plasmid encoding 8 flagellar proteins: FlhA, FlhB, FliF, FliG-His, FliO, FliP, HA-FliQ, and FliR-FLAG (S10A Fig). Immunoblotting revealed that they were expressed in the *Salmonella* SJW1368 strain (S10B Fig). The membranes were solubilized by 1% DDM, and then the proteins were purified by Ni affinity chromatography. Only FlhA and FliF copurified with His-FliG from DDM-solubilized membranes of the cells expressing FlhA, FlhB, FliF, FliG-His, FliO, FliP, HA-FliQ, and FliR-FLAG (Fig 5A), indicating that the FliO/FliP/FliR-FLAG/FlhB complex and HA-FliQ dissociate from the FlhA/FliF/FliG complex. The FlhA/FliF/FliG complex was further purified by SEC, and then the main peak fraction containing FlhA, FliF, and FliG was analyzed by EM with negative staining. Many MS rings were observed in the pooled fractions (Fig 5B), indicating that FlhA associates with the MS ring.

**Fig 6:**
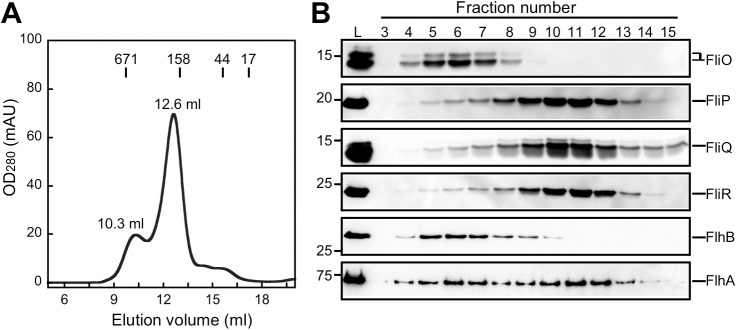
Effect of lauryl maltose neopentyl glycol (LMNG) on the interactions of the FliP_6_ ring with other export gate proteins. (A) Elution profiles of the FlhA/FlhB/FliO/FliP/FliQ/FliR complex from a Superdex 200 10/300 column equilibrated with 20 mM Tris-HCl, pH 8.0, 150 mM NaCl, 2 mM EDTA, 5% glycerol, and 0.01% LMNG. Membrane fractions were prepared from SJW1368 expressing FlhA, FlhB, FliO, His-FliP, HA-FliQ, and FliR-FLAG and were solubilized by 1% LMNG. Then, the protein complex was purified by Ni affinity chromatography, followed by size exclusion chromatography (SEC). (B) Immunoblotting of elution fractions from A, using anti-FliO (first row), anti-His (second row), anti-HA (third row), anti-FLAG (fourth row), anti-FlhB_C_ (fifth row), or anti-FlhA_C_ (sixth row) antibody. (Note: The C-terminal cytoplasmic domain of FlhB undergoes autocleavage between conserved Asp-269 and Pro270 residues [2–4], and hence, the molecular size of the FlhB band recognized by polyclonal anti-FlhB_C_ antibody is smaller than that of full-length FlhB). These proteins treated with LMNG showed slightly distinct running behavior on SDS gels compared to those with n-dodecyl β-D-maltoside (DDM), presumably due to the detergent effect. The lane marked L represents the material loaded onto the SEC column.

It has been shown by in vivo photo-crosslinking experiments that SpaQ interacts with SpaP and SpaR in the final assembled export gate complex. However, an assembly intermediate complex isolated from DDM-solubilized membranes contains only SpaP and SpaR, which may be due to loss of SpaQ in response to DDM extraction [33–35]. FliQ is an essential export component of the flagellar type III export apparatus [38]. FlhA requires FliQ for efficient assembly of the FlhA ring structure inside the MS ring [8], raising the possibility that DDM affects interactions of FlhA and FliQ with other export gate proteins. To test this, we solubilized the membrane fractions of *Salmonella* cells expressing FlhA, FlhB, FliO, His-FliP, HA-FliQ, and FliR-FLAG by 1% lauryl maltose neopentyl glycol (LMNG) instead of DDM and purified it by Ni affinity chromatography, followed by SEC with a Superdex 200 10/300 column (Fig 6). The SEC elution profile of the FlhA/FlhB/FliO/FliP/FliQ/FliR complex showed 2 distinct peaks (Fig 6A). The first peak (10.3 ml, Fig 6A) mainly contained FliO, FlhB, and FlhA along with a much smaller amount of His-FliP (Fig 6B). Since FlhB copurified with His-FliP, FliO, and FliR-FLAG upon membrane solubilization by DDM (Fig 5A and S8 Fig), we suggest that LMNG weakens the interactions of FliO and FlhB with FliP and FliR. The second peak (12.6 ml, Fig 6A) mainly contained FliP, FliQ, FliR, and FlhA (Fig 6B), indicating that FliQ and FlhA bind to the FliP/FliR complex, although some of the FlhA molecules are dissociated from the complex along with FliO and FlhB.

## Discussion

The export gate complex is composed of 5 highly conserved transmembrane proteins—namely, FlhA, FlhB, FliP, FliQ, and FliR—although the transmembrane protein FliO is required for efficient assembly of the export gate in *Salmonella* [6,7]. FliP and FliR are postulated to form a core structure for the assembly of other export gate proteins [31,32]. Recently, it has been reported that SpaP of the *Salmonella* SPI-1 T3SS forms a homopentamer [34]. In contrast to SpaP, we showed that *St*-FliP forms a homohexamer with a diameter of about 10 nm (Fig 2). The F137A, F150A, and E178A substitutions in FliP_P_ interfered with FliP_6_ ring formation (S2 Fig) and reduced the export function considerably (Fig 1D). Therefore, we suggest that the FliP_6_ ring is a functional unit in the export gate complex and that Phe-137, Phe-150, and Glu-178 are responsible for FliP_6_ ring formation. Thus, it seems that the core structure of the flagellar export gate complex is somewhat different from that of the T3SS of pathogenic bacteria. However, 3,780 of the 11,736 FliP ring particles analyzed were assigned to the 5-fold rotational symmetry (S3E Fig), raising the possibility that FliP forms a homopentamer in a way similar to the SpaP_5_ ring.

We have solved the crystal structure of the *Tm-*FliP_P_ tetramer and have built 2 *St-*FliP_P_ dimer models on the basis of the *Tm-*FliP_P_ structure (Fig 3). We have also mapped 3 functionally important residues—namely, Phe-137, Phe-150, and Glu-178 —onto the *St-*FliP_P_ model. Although the A–B and A–C dimers are found in the crystal, the A–B dimer seems to be the dimer unit of the ring structure, as supported by photo-crosslinking experiments (Fig 4). However, since Phe-137 and Glu-178, which are involved in A–C dimer formation (Fig 3E) and are located on the bottom surface close to the rectangular corner of the A–B dimer (Fig 3F), are required for the ring formation and export function (Fig 1 and S2 Fig), they are likely to be involved in the ring formation by connecting the dimers.

The FliP(R143H) and FliP(F190L) mutations, which are located in FliP_P_ and TM-3 of FliP, respectively, improve motility of the ∆*fliO* mutant to some extent [6,7]. This suggests the presence of FliO–FliP interaction. Here, we provided direct evidence that FliP binds to FliO (Figs 1 and 4). Negatively stained EM analysis revealed that FliO forms a 5-nm ring structure with 3 clamp-like structures that bind to the FliP_6_ ring (Fig 2 and S4 Fig). Photo-crosslinking experiments revealed direct interactions of FliO with FliP-TM1 and FliP_P_ (Fig 4). Overexpression of FliP restored motility of the ∆*fliO* mutant to the wild-type level (S5 Fig), suggesting that the FliO ring complex does not exist in the final structure of the export gate complex. In agreement with this, FliO homologues are absent in nonflagellar T3SSs [5]. Therefore, we propose that the FliO ring complex acts as a scaffold to catalyze FliP_6_ ring formation and that the interactions of FliO with FliP may induce structural rearrangements of the FliP_P_ dimer to facilitate FliP_6_ ring formation. Because Arg-143 is located on the rectangular corner surface of the A–B dimer and near Phe-137 and Glu-178 (Fig 3F), we suggest that the R143H and F190L mutations in FliP increase the probability of FliP_6_ ring formation in the absence of FliO. Since the virulence-associated T3SS apparatus does not have the FliO homologue, we assume that FliP homologues may have a self-scaffolding function to facilitate their own ring formation.

The export gate complex of the SPI1-T3SS contains 5 SpaP molecules, 1 SpaQ, 1 SpaR, 1 SpaS and 9 InvA subunits [33–35]. SpaQ, SpaR, and SpaS assemble onto the SpaP pentamer and closely interact with each other [34]. Here we showed that FliR and FlhB copurified with the FliO/FliP ring complex when isolated from DDM-solubilized membrane of *Salmonella* cells expressing FlhA, FlhB, FliO, FliP, FliQ, and FliR (Fig 5 and S9 Fig). Relative band intensities of FliP, FliR, and FlhB in the FlhB/FliO/FliP/FliR complex allowed us to roughly estimate that the complex contains 6 copies of FliP, 2 copies of FliR, and 2 copies of FlhB. This is in good agreement with 2 sets of previous experimental data that FlhB forms a homodimer in the basal body [39] and that FliR and FlhB associate with each other in a 1 to 1 fashion [30]. When we used LMNG as a detergent instead of DDM, both FlhA and FliQ coeluted with the FlhB/FliO/FliP/FliR complex from a Ni-NTA column (Fig 6B, the lane marked L), indicating that they bind to the FlhB/FliO/FliP/FliR complex. In contrast to the complex solubilized by DDM, FliO and FlhB dissociated from the complex during SEC. However, FlhA and FliQ were associated with the FliP/FliR complex, although some of the FlhA molecules were dissociated from the complex along with FliO and FlhB (Fig 6B). Taken all together, we suggest that FlhA, FlhB, FliQ, and FliR assemble onto the FliP_6_ ring in complex with FliO to form the export gate complex.

**Fig 7:**
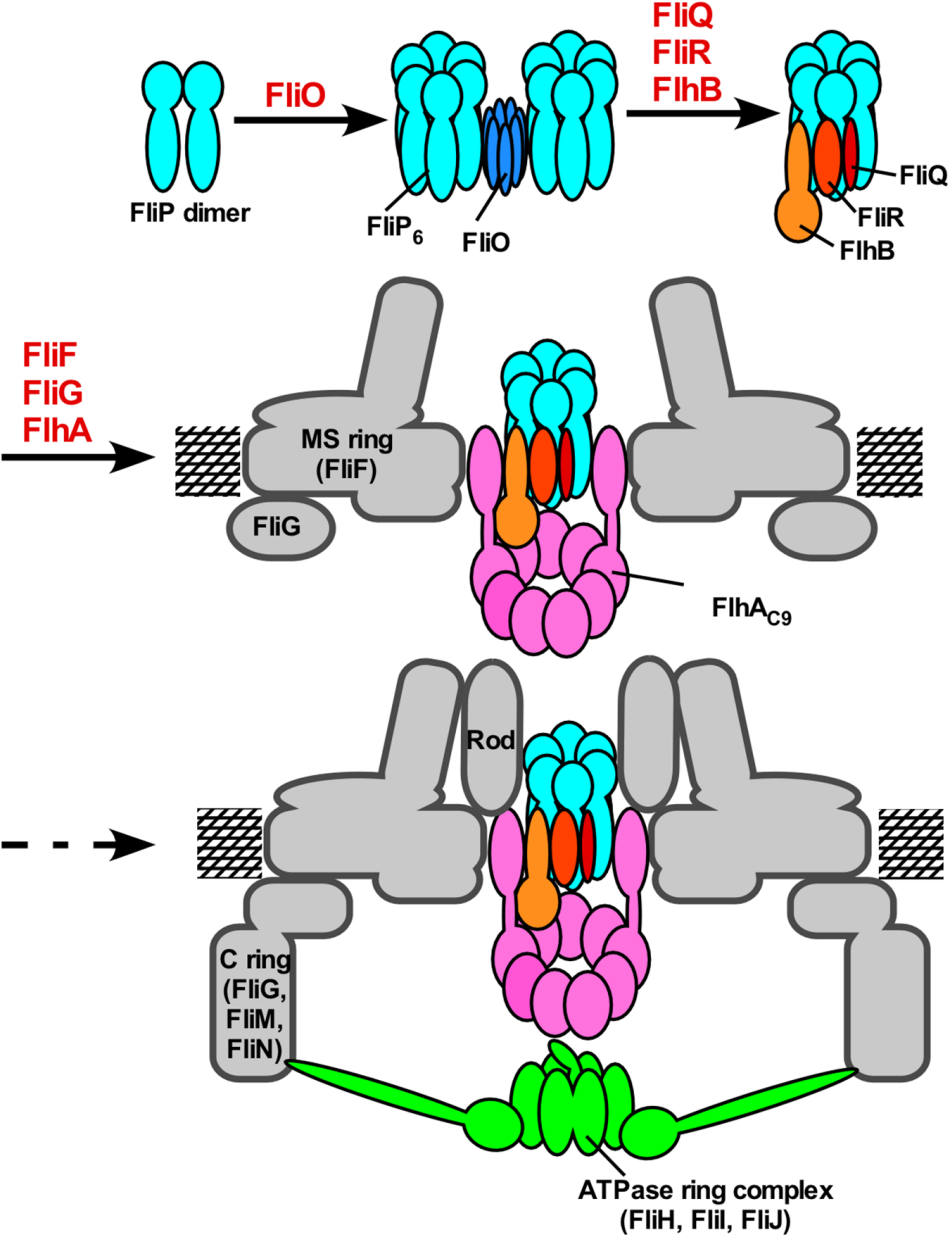
Model for the assembly process of the flagellar type III export apparatus. The export apparatus is composed of a transmembrane export gate complex made of FlhA, FlhB, FliP, FliQ, and FliR and a cytoplasmic ATPase ring complex consisting of FliH, FliI, and FliJ. The FliP dimers form a homohexamer with the help of the FliO complex, followed by the assembly of FliQ, FliR, and FlhB and finally of FlhA during MS ring formation in the cytoplasmic membrane. Then, the FliM/FliN complex binds to FliG to form the C ring on the cytoplasmic face of the MS ring. Finally, the FliH/FliI/FliJ ATPase ring complex is formed at the flagellar base through interactions of FliH with FlhA and FliN [2–4], allowing export substrates to go into the central cavity of the FliP_6_ ring complex.

When FliF, FliO, FliP, FliQ, FliR, FlhA, and FlhB were coexpressed with His-FliG (S10 Fig), only FlhA copurified with the FliF-FliG ring complex from the DDM-solubilized membrane (Fig 5), indicating that FlhA directly associates with the MS ring. This is in agreement with a previous report that a *Salmonella fliF(*∆*174-175)* mutant gives rise to extragenic suppressor mutations in FlhA_TM_ [27]. It has been shown that FlhA forms a homononamer inside the MS ring [8,13,14] and that the assembly of FlhA to the MS ring is required for FliO, FliP, FliQ, and FliR [8]. Since we found that some FlhA molecules associate with the FliP/FliQ/FliR complex (Fig 6B), we propose that the assembly of the export gate complex begins with FliP_6_ ring formation with the help of the FliO scaffold, followed by the assembly of FliQ, FliR, and FlhB and finally of 9 FlhA molecules during MS ring formation in the cytoplasmic membrane (Fig 7).

In summary, we have presented direct evidence that FliP forms a homohexamer with the help of the FliO complex and that FliP_P_–FliP_P_ and FliP_P_–FliO interactions are required for efficient FliP_6_ ring formation. Our most important findings are that FliP_6_ ring formation is essential for flagellar type III protein export (Fig 1). Since there are many structural and functional similarities between the flagellar and T3SS proteins, the periplasmic domain of FliP homologues of the T3SSs could be a good target for inhibitors specific for bacterial infection.

## Materials and methods

### Bacterial strains, plasmids, DNA manipulations, and media

Bacterial strains and plasmids used in this study are listed in S1 Table. DNA manipulations, site-directed mutagenesis, and DNA sequencing were carried out as described previously [40]. L-broth (LB) and soft tryptone agar plates were used as described before [38,41]. The 2×YT medium contained 1.6% (w/v) Bacto-tryptone, 1.0% (w/v) Bacto-yeast extract, and 0.5% (w/v) NaCl.

### Purification of FliP alone and FliP in complex with FliO

For expression and purification of FliP, *Salmonella* SJW1368 cells harboring pKY069 were grown in 2×YT medium containing 100 μg ml^-1^ ampicillin at 30°C until the cell density had reached an OD_600_ of about 0.4–1.0 and then were incubated at 16°C for another 24 h. Cells were harvested by centrifugation (6,400 g, 10 min, 4°C) and stored at −80°C. The cells were thawed, resuspended in 20 mM Tris-HCl, pH 8.0, 3 mM EDTA, and disrupted by sonication. The cell lysates were centrifuged (20,000 g, 15 min, 4°C) to remove cell debris. The supernatants were ultracentrifuged (110,000 g, 1 h, 4°C). The harvested membranes were stored at −80°C. The membranes were solubilized in 50 mM Tris-HCl, pH 8.0, 300 mM NaCl, 5% glycerol, 20 mM imidazole, and 1% DDM at 4°C for 30 min and ultracentrifuged (110,000 g, 30 min, 4°C) to remove the insoluble membrane fractions. Solubilized membranes were loaded onto a Ni-NTA agarose column (GIAGEN) and washed extensively with 50 mM Tris-HCl, pH 8.0, 300 mM NaCl, 5% glycerol, 20 mM imidazole, and 0.1% DDM. Proteins were eluted with a 50–400 mM imidazole gradient. Fractions containing His-FliP were concentrated and further purified by SEC with a Superdex 200 10/300 column (GE Healthcare) equilibrated with 20 mM Tris-HCl, pH 8.0, 300 mM NaCl, 2 mM EDTA, 5% glycerol, and 0.1% DDM.

For purification of the FliO/His-FliP complex, the membrane fractions were prepared from the SJW1368 cells carrying pKY070 or its mutant variant plasmids in a way similar to His-FliP. After solubilization with 1% DDM, the FliO/His-FliP complex and its mutant variants were purified by Ni-NTA chromatography, followed by SEC with a Superdex 200 10/300 column equilibrated with 20 mM Tris-HCl, pH 8.0, 300 mM NaCl, 2 mM EDTA, 5% glycerol, and 0.1% DDM.

### EM and image processing

Samples were applied to carbon-coated copper grids and negatively stained with 1.0% (w/v) uranyl acetate. Micrographs were recorded at a magnification of ×50,000 with a JEM-1011 transmission electron microscope (JEOL, Tokyo, Japan) operated at 100 kV. To carry out 2D class averaging of the FliP ring structure and the FliO/FliP complex, 11,736 and 1,961 particle images, respectively, were boxed out with e2boxer.py [42], aligned, classified, and averaged using the e2refine2d.py program [42]. To estimate the stoichiometry of the FliP ring, a typical class averaged image was converted from cartesian to polar coordinates, and then the autocorrelation function was calculated. The rotational symmetry was analyzed from Fourier transformation of the autocorrelation function. To carry out 2D class averaging of the FliO structure, 14,915 particle images were boxed out with e2boxer.py [42], aligned, classified, and averaged using the RELION program [43].

### Multiple sequence alignment

Multiple sequence alignment was performed by CLUSTAL-Ω (http://www.ebi.ac.uk/Tools/msa/clustalo/).

### Purification and crystallization of *Tm-*FliP_P_

Details of the expression, purification, and crystallization of *Tm-*FliP_P_ have been described previously [37].

### Data collection and structure determination

X-ray diffraction data were collected at the synchrotron beamline BL41XU in SPring-8 (Harima, Japan) with the approval of the Japan Synchrotron Radiation Research Institute (JASRI) (Proposal No. 2013B1305). Details of the X-ray data collection and processing are described previously [37]. Crystals were frozen in liquid nitrogen and mounted in nitrogen gas flow at 100 K. The X-ray diffraction data were collected on an MX225HE CCD detector (Rayonix), were processed with iMOSFLM [44], and were scaled using SCALA [45]. The statistics of the diffraction data have been described previously [37]. The experimental phase was calculated using the SAD data of the Se-Met derivative with the program Phenix [46]. The atomic model was built with Coot [47] and refined to 2.4 Å with Phenix [46] against the native crystal data that showed the best resolution limit. The refinement R factor and the free R factor were converged to 21.5% and 26.2%, respectively. The Ramachandran plot indicated that 96.5% and 3.5% residues were located in the most favorable and allowed region, respectively. Structural refinement statistics are summarized in S2 Table. The atomic coordinates have been deposited in PDB under the accession code 5H72.

### Homology modeling

The structure of *St-*FliP_P_ was modeled by using SWISS-MODEL [48]. The amino acid sequence from Ile-121 to Phe-183 of *St-*FliP was used for the target sequence, and the crystal structure of *Tm-*FliP_P_ was used for a template to construct the homology model.

### Motility assay

Fresh transformants were inoculated onto soft tryptone agar plates containing 100μg ml^-1^ ampicillin and 0.2% arabinose and incubated at 30°C. At least 7 independent measurements were carried out.

### Secretion assays

Details of sample preparation have been described previously [49]. After SDS-PAGE, immunoblotting with polyclonal anti-FlgD, anti-FlgE, anti-FlgK, or anti-FlgL antibody was carried out as described previously [38]. Detection was done with an ECL immunoblotting detection kit (GE Healthcare). At least 3 independent experiments were carried out.

### In vivo photo-crosslinking

*E*. *coli* BL21 (DE3) cells were transformed with a low-copy-number pTACO10-based plasmid [35] and the amber suppressor plasmid pSup-pBpa [50]. The transformed BL21 (DE3) cells were cultured at 37°C in LB containing 10 μg ml^-1^ chloramphenicol and 25 μg ml^-1^ kanamycin. Cultures were supplemented with 500 μM rhamnose to induce the expression of FliO/FliP-FLAG/FliQ/FliR, FliO/FliP-FLAG, or FliO-FLAG/FliP-HA from the pTACO10-based plasmid. Additionally, the cultures were supplemented with pBPA to a final concentration of 1 mM and afterwards incubated for 5.5 h. Two OD units of bacterial cells were harvested and washed once with 1 ml cold PBS (8 g of NaCl, 0.2 g of KCl, 3.63 g of Na_2_HPO_4_ 12H_2_O, 0.24 g of KH_2_PO_4_, pH 7.4 per liter). Cells were resuspended in 1 ml PBS and transferred into 6-well cell culture dishes for 30 min UV irradiation (λ = 365 nm) using a UV transilluminator table (UVP).

Two OD units of bacterial lysates of *E*. *coli* were resuspended in 750 μl buffer K (50 mM triethanolamine, pH 7.5, 250 mM sucrose, 1 mM EDTA, 1 mM MgCl_2_, 10 μg/ml DNAse, 2 mg/ml lysozyme, 1:100 protease inhibitor cocktail) and incubated for 30 min on ice. Samples were bead milled, and beads, unbroken cells, and debris were removed by centrifugation (10,000 g, 10 min, 4°C). Crude membranes contained in the supernatant were precipitated by ultracentrifugation using a Beckman TLA 55 rotor (100,000 g, 45 min, 4°C). Pellets containing crude membranes were frozen until use. For protein detection, samples were subjected to SDS-PAGE using SERVAGel TG PRiME 8%–16% or SERVAGel TG PRiME 12% precast gels, transferred onto a PVDF membrane (Bio-Rad), and probed with M2 anti-FLAG antibody (Sigma). Anti-mouse IgG DyLight 800 (Thermo Fisher) was used as a secondary antibody. Scanning of the PVDF membrane and image analysis were performed with a Li-Cor Odyssey system and Image Studio 2.1.10 (Li-Cor).

### Copurification assays

His-FliP/FliR-FLAG, His-FliP/HA-FliQ/FliR-FALG, FliO/His-FliP/FliR-FLAG, FliO/His-FliP/HA-FliQ/FliR-FLAG, FliO/His-FliP/HA-FliQ/FliR-FLAG/FlhB/FlhA, or FliO/ FliP/HA-FliQ/FliR-FLAG/FlhB/FlhA/FliF/His-FliG was expressed in *Salmonella* SJW1368 cells harboring a pTrc99A-based plasmid, solubilized by 1% DDM or 1% LMNG, and purified by Ni-NTA chromatography, followed by FLAG affinity chromatography. Proteins were eluted from anti-FLAG affinity gels (Sigma) with 100 μg ml^-1^ of FLAG peptide (Sigma). The His-FliP/FliR-FLAG, FliO/His-FliP/FLAG-FliR, and FliO/His-FliP/FLAG-FliR/FlhB complexes were further purified by SEC.

## Supporting information

S1 Fig Topological model of *St*-FliP. (A) St-FliP is a transmembrane membrane protein with a cleavable signal peptide (SP) at its N-terminus. The signal peptide of FliP (Met-1 to Gln-22) is cleaved during its membrane insertion [36]. The mature form of St-FliP has four transmembrane (TM) helices and a periplasmic domain (FliP_P_) between TM-2 and TM-3. (B) Amino acid sequence of St-FliP. Conserved residues in FliP_P_ are highlighted in red. Colored regions are matched in A and B.
(TIF)

S2 Fig Purification of FliP and the FliO/FliP complex by size exclusion chromatography. (A) Elution profiles of FliP, the FliO/FliP complex, the FliO/FliP(F137A) complex, the FliO/FliP(F150A) complex and the FliO/FliP(E178A) complex from a Superdex 200 10/300 column equilibrated with 20 mM Tris-HCl pH 8.0, 150 mM NaCl, 2 mM EDTA, 5% glycerol and 0.1% DDM. The elution positions are shown by peaks 1, 2, 3 and 4. Peak fractions of molecular mass markers (670 kDa, 158 kDa and 44 kDa) are shown. (B) Representative negatively stained EM images of each peak fraction. Scale bar shows 50 nm. Peaks 1, 2, 3 and 4 contained the FliO/FliP_6_ ring complex, the FliP_6_ ring, the FliO ring and the FliP dimer, respectively.
(TIF)

S3 Fig Stoichiometry of the FliP ring. (A) A representative reference-free 2D class average images of the FliP ring calculated from e2refine2d.py (EMAN2). (B) Polar coordinates conversion from area sandwiched by two green dashed lines in A. (C) Auto-correlation plots calculated from the image obtained by polar coordinates conversion. (D) Result of reference-free 2D class average images of the FliP ring calculated from e2refine2d.py (EMAN2). The number of particles for each class is shown in the top right corner. (E) Histogram of the number of particles between three and eight symmetries, resulted from auto-correlation analysis.
(TIF)

S4 Fig 2D class averaging of the FliO ring complex and the FliO/FliP complex. (A) Reference-free 2D class average images of the FliO ring complex calculated from RELION. (B) Reference-free 2D class average images of the FliO/FliP complex calculated from e2refine2d.py (EMAN2). The number of particles for each class is shown in the top right corner.
(TIF)

S5 Fig Multicopy effect of FliP on motility of a *Salmonella* ∆*fliO* mutant. Motility of TH10548 transformed with pTrc99A (V), pKY073 (FliO), pKY074 (*P_ara_*-HA-FliP) or pKY010 (*P_lac_*-HA-FliP) in soft agar (upper panel). Immunoblotting, using polyclonal anti-FliOC (middle panel) or monoclonal anti-HA antibody (lower panel), of whole cell lysates prepared form the above strains. (Note: the level of HA-FliP expressed from the pBAD24 vector was not detected at all.).
(TIF)

S6 Fig Purification of the FliO ring complex. (A) Stability of the FliO/FliP ring complex during storage at 4°C. The FliO/FliP complex stored at 4°C for 1 day was run on a Superdex 200 10/300 column. SDS-PAGE of elution fractions. Molecular mass markers (kDa) are shown on the left. (B) SEC analysis of the FliO complex. Fractions containing the FliO complex was pooled and analyzed by SDS-PAGE with CCB staining.
(TIF)

S7 Fig Surface properties of the A–B and A–C dimers of *Tm*-FliP_P_. (A) A–B dimer form of Tm-FliP_P_ (B) A–C dimer form of Tm-FliP_P_. Residues involved in the dimer interaction are indicated with ball-and-stick representation. (C), (D) A–B dimer interface. (F), (G) A–C dimer interface. The A subunit is shown in surface representation painted with yellow and white for hydrophobic and the other residues, respectively. The B and C subunits are shown by Cα-trace colored with pink in (C) and (F), respectively. Residues involved in the dimer interaction are labeled in (D) and (G). (E), (H) The A–B (E) and A–C dimers (H) are viewed from the opposite side of both C-termini (viewed from the top of the model in (C) and in (F), respectively). The hydrophobic [same color as (C)] and the electrostatic potential (red, negative; blue, positive) surfaces are shown in left and right panels, respectively. The Cα-trace of the dimer is in the middle panel.
(TIF)

S8 Fig Surface properties of the A–B and A–C dimers of *St*-FliP_P_. (A), (B) A–B dimer interface. (D), (E) A–C dimer interface. The A subunit is shown in surface representation painted with yellow and white for hydrophobic and the other residues, respectively. The B and C subunits are shown by Cα-trace colored with pink in (A) and (D), respectively. Residues involved in the dimer interaction are labeled in (B) and (E). (C), (F) The A–B (C) and A–C (F) dimers viewed from the opposite side of both C-termini (viewed from the top of the model in (A) and in (D), respectively). The hydrophobic (same color as (A)) and the electrostatic potential (red, negative; blue, positive) surfaces are shown in left and right panels, respectively. The Cα-trace of the dimer is in the middle panel.
(TIF)

S9 Fig Interactions of the FliP_6_ ring with other export gate components. (A) Co-purification assays by anti-FLAG M2 affinity chromatography. Membranes were prepared from SJW1368 expressing His-FliP and FliR (P/R), His-FliP, FliQ and FliR (P/Q/R), FliO, His-FliP and FliR (O/P/R), FliO, His-FliP, FliQ and FliR (O/P/Q/R), or FlhA, FlhB FliO, His-FliP, FliQ and FliR (O/P/Q/R/B/A) and solubilized by 1% DDM, followed by Ni affinity chromatography and finally anti-FLAG M2 affinity chromatography. “Input” indicates pooled fractions after Ni affinity chromatography. “Flow through” and “Elution” indicate the flow through and elution fractions, respectively. Each fraction obtained by anti-FLAG M2 affinity chromatography was analyzed by SDS-PAGE with CBB staining. Molecular mass markers (kDa) are shown on the left. (B) Analysis of purified FliP/FliR by SEC with a Superose 6 10/300 column. SDS-PAGE of pooled fractions shown by grey line. Representative negatively stained EM image of the peak fraction. Scale bar shows 50 nm.
(TIF)

S10 Fig Co-expression system of flagellar type III export gate proteins. (A) Schematic diagram of plasmid construction of pKY079. (B) Expression of FlhA, FlhB, FliF, FliG-His, FliO, FliP, HA-FliQ and FliR-FLAG as judged by immunoblotting with anti-FlhA_C_, anti-FlhB_C_, anti-FliF, anti-His, anti-FliO_C_, anti-FliP_P_, anti-HA and anti-FLAG antibodies, respectively.
(TIF)

S1 Table Strains and plasmids used in this study. (DOCX)

S2 Table X-ray refinement statistics. (DOCX)

## Abbreviations

pBPA: *p*-benzoyl-phenylalanine
CBB: Coomassie Brilliant Blue
CM: cytoplasmic membrane
DDM: n-dodecyl β-D-maltoside
EM: electron microscopy
LMNG: lauryl maltose neopentyl glycol
NTA: nitrilotriacetic acid
PDB: Protein Data Bank
SEC: size exclusion chromatography
T3SS: type III secretion system
TM: transmembrane
WT: wild type
