# Supplementary figures and images for: Assembly and stoichiometry of the core structure of the bacterial flagellar type III export gate complex
Source: PLoS Biol. 2017 Aug 3;15(8):e2002281. doi: 10.1371/journal.pbio.2002281 (PMC5542437; doi:10.1371/journal.pbio.2002281)

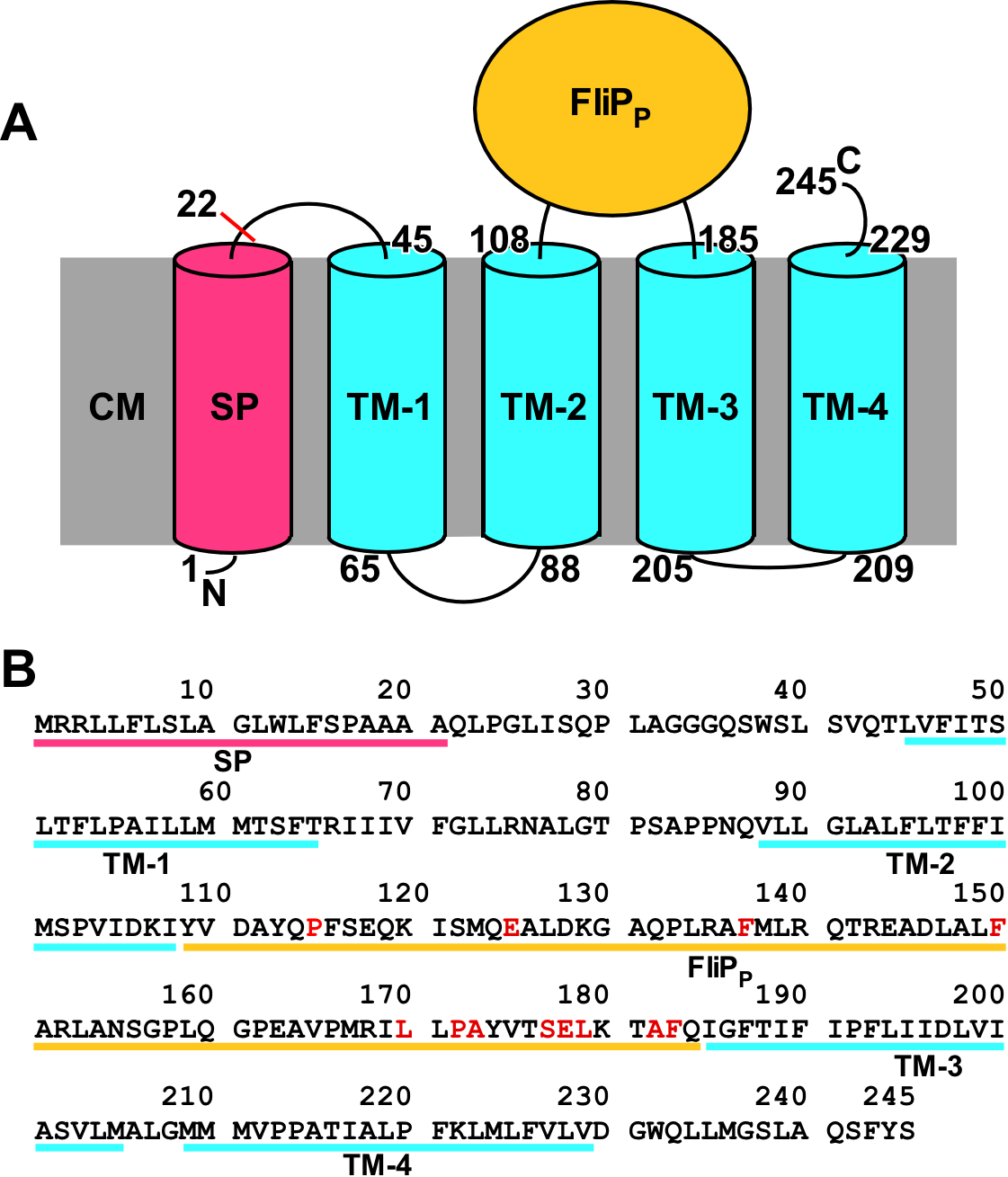

Supplement: S1 Fig — (A) St-FliP is a transmembrane membrane protein with a cleavable signal peptide (SP) at its N-terminus. The signal peptide of FliP (Met-1 to Gln-22) is cleaved during its membrane insertion [36]. The mature form of St-FliP has four transmembrane (TM) helices and a periplasmic domain (FliPP) between TM-2 and TM-3. (B) Amino acid sequence of St-FliP. Conserved residues in FliPP are highlighted in red. Colored regions are matched in A and B. (TIF) [file pbio.2002281.s001.tif]

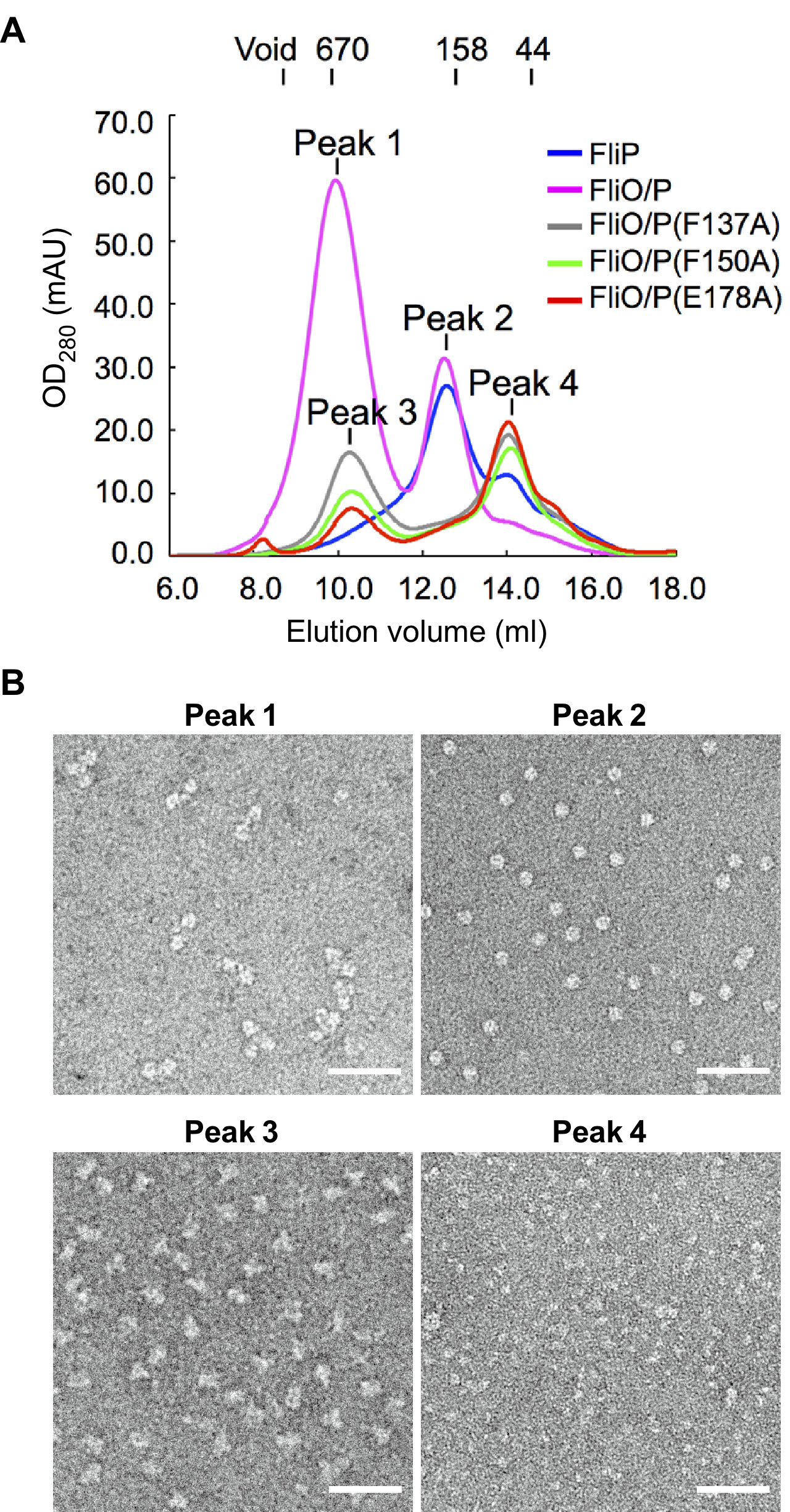

Supplement: S2 Fig — (A) Elution profiles of FliP, the FliO/FliP complex, the FliO/FliP(F137A) complex, the FliO/FliP(F150A) complex and the FliO/FliP(E178A) complex from a Superdex 200 10/300 column equilibrated with 20 mM Tris-HCl pH 8.0, 150 mM NaCl, 2 mM EDTA, 5% glycerol and 0.1% DDM. The elution positions are shown by peaks 1, 2, 3 and 4. Peak fractions of molecular mass markers (670 kDa, 158 kDa and 44 kDa) are shown. (B) Representative negatively stained EM images of each peak fraction. Scale bar shows 50 nm. Peaks 1, 2, 3 and 4 contained the FliO/FliP6 ring complex, the FliP6 ring, the FliO ring and the FliP dimer, respectively. (TIF) [file pbio.2002281.s002.tif]

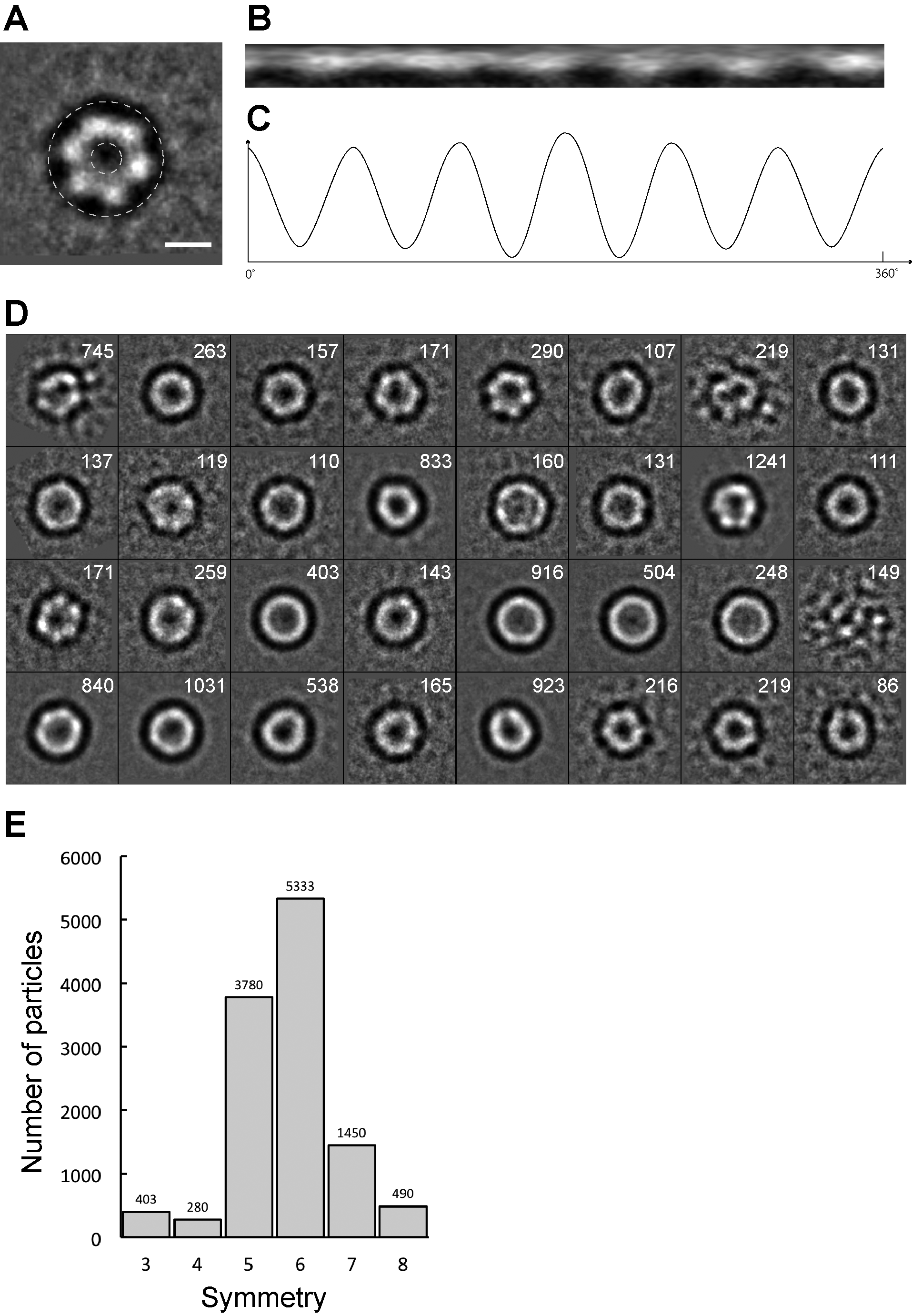

Supplement: S3 Fig — (A) A representative reference-free 2D class average images of the FliP ring calculated from e2refine2d.py (EMAN2). (B) Polar coordinates conversion from area sandwiched by two green dashed lines in A. (C) Auto-correlation plots calculated from the image obtained by polar coordinates conversion. (D) Result of reference-free 2D class average images of the FliP ring calculated from e2refine2d.py (EMAN2). The number of particles for each class is shown in the top right corner. (E) Histogram of the number of particles between three and eight symmetries, resulted from auto-correlation analysis. (TIF) [file pbio.2002281.s003.tif]

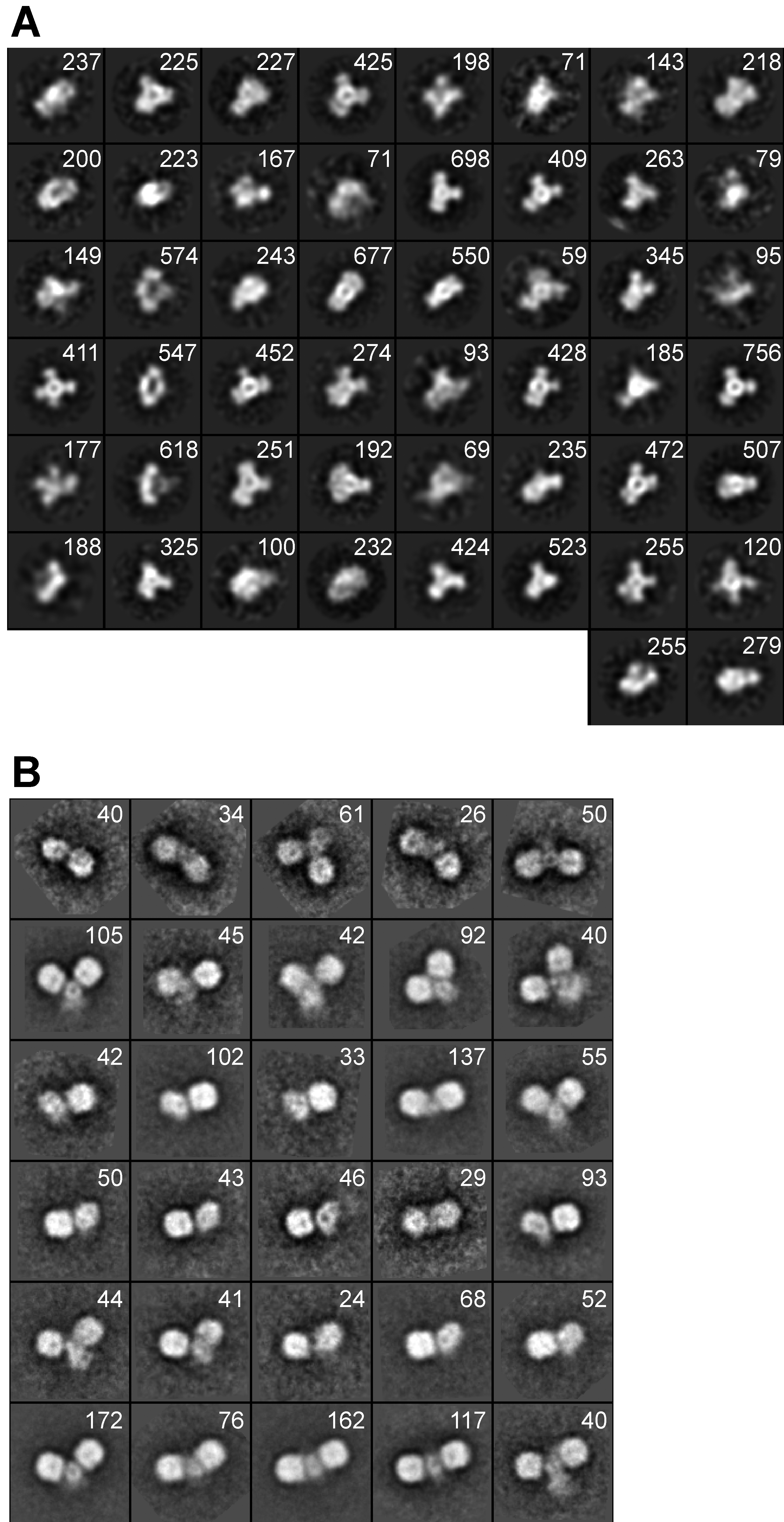

Supplement: S4 Fig — (A) Reference-free 2D class average images of the FliO ring complex calculated from RELION. (B) Reference-free 2D class average images of the FliO/FliP complex calculated from e2refine2d.py (EMAN2). The number of particles for each class is shown in the top right corner. (TIF) [file pbio.2002281.s004.tif]

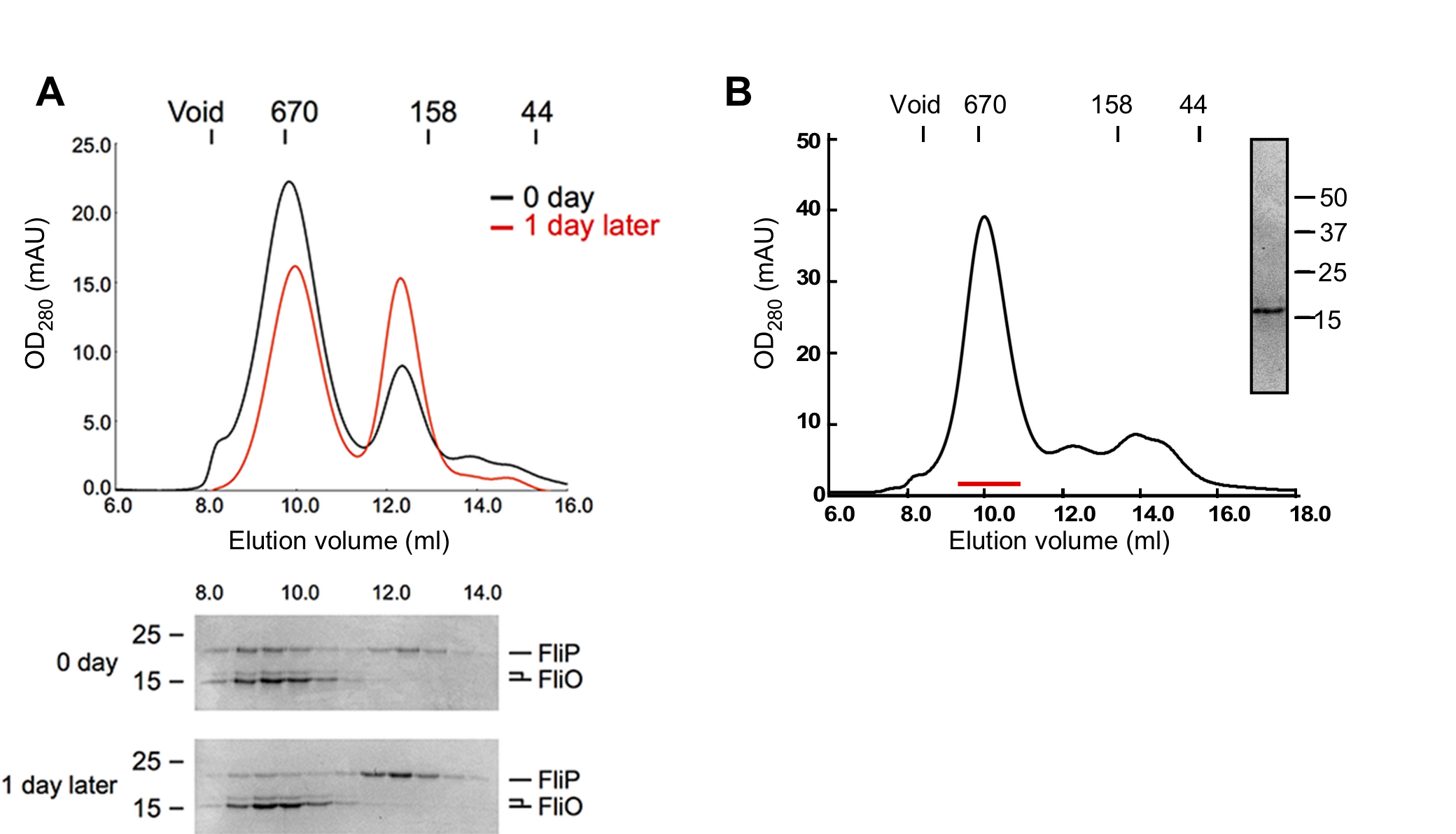

Supplement: S6 Fig — (A) Stability of the FliO/FliP ring complex during storage at 4°C. The FliO/FliP complex stored at 4°C for 1 day was run on a Superdex 200 10/300 column. SDS-PAGE of elution fractions. Molecular mass markers (kDa) are shown on the left. (B) SEC analysis of the FliO complex. Fractions containing the FliO complex was pooled and analyzed by SDS-PAGE with CCB staining. (TIF) [file pbio.2002281.s006.tif]

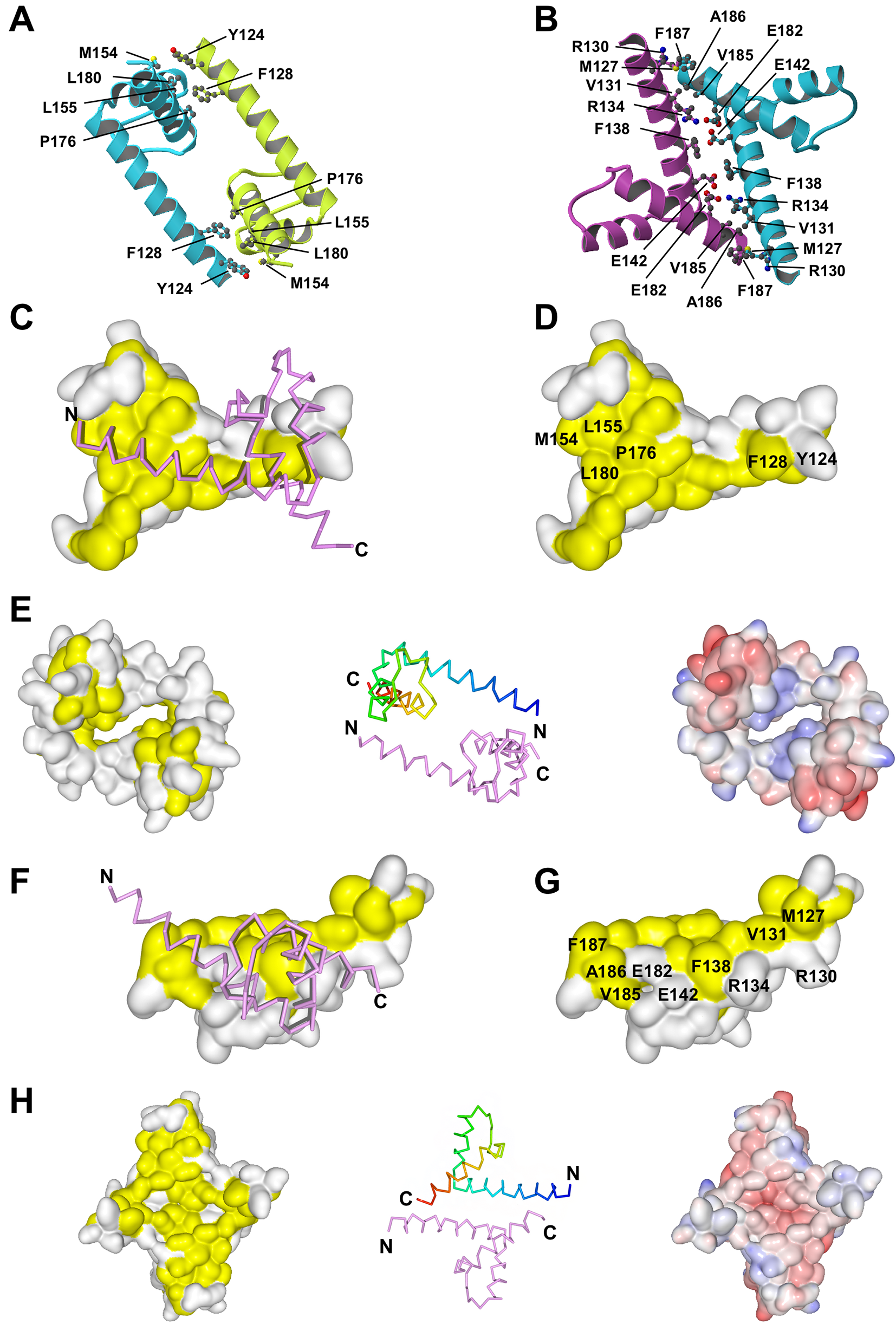

Supplement: S7 Fig — (A) A–B dimer form of Tm-FliPP (B) A–C dimer form of Tm-FliPP. Residues involved in the dimer interaction are indicated with ball-and-stick representation. (C), (D) A–B dimer interface. (F), (G) A–C dimer interface. The A subunit is shown in surface representation painted with yellow and white for hydrophobic and the other residues, respectively. The B and C subunits are shown by Cα-trace colored with pink in (C) and (F), respectively. Residues involved in the dimer interaction are labeled in (D) and (G). (E), (H) The A–B (E) and A–C dimers (H) are viewed from the opposite side of both C-termini (viewed from the top of the model in (C) and in (F), respectively). The hydrophobic [same color as (C)] and the electrostatic potential (red, negative; blue, positive) surfaces are shown in left and right panels, respectively. The Cα-trace of the dimer is in the middle panel. (TIF) [file pbio.2002281.s007.tif]

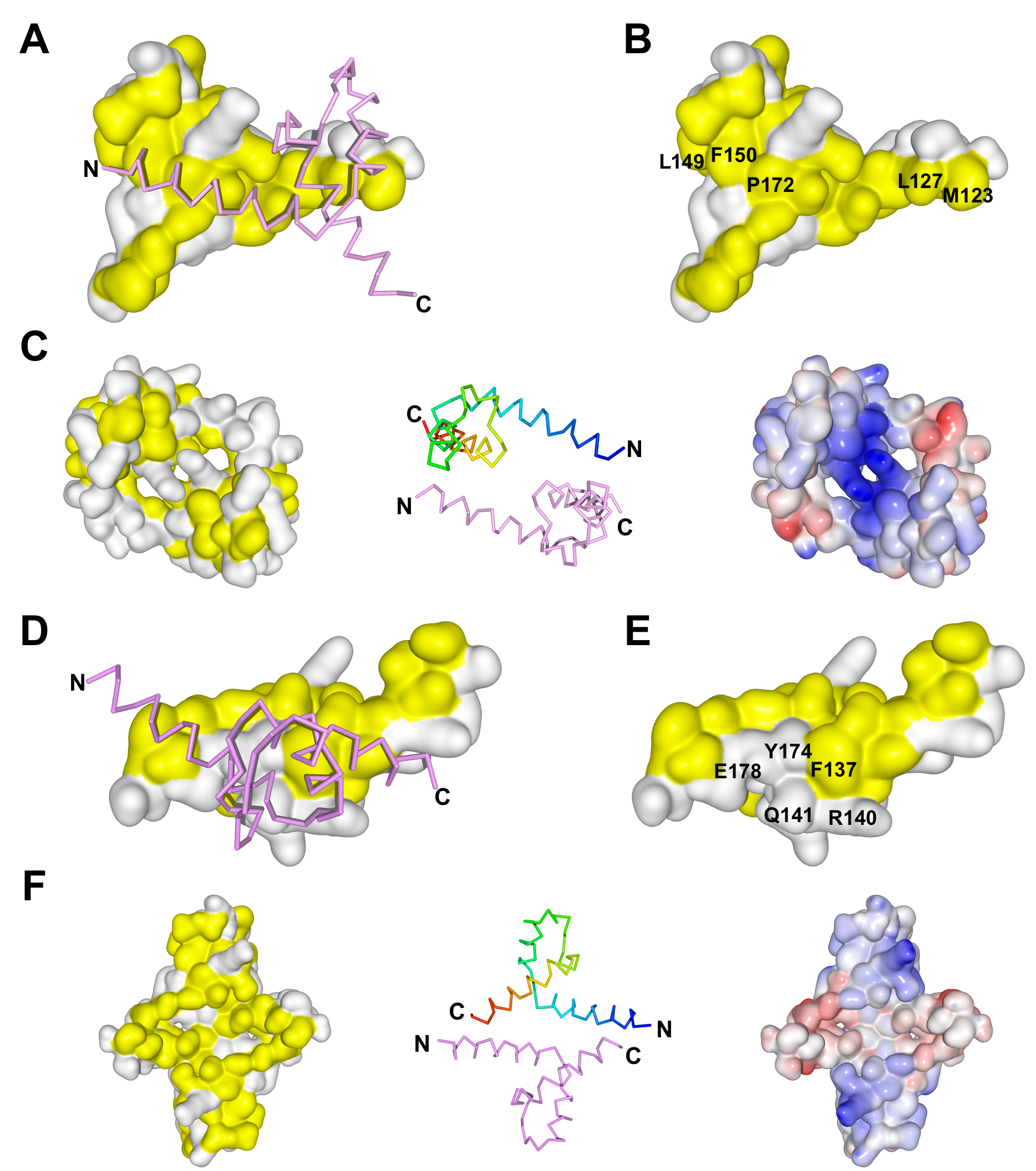

Supplement: S8 Fig — (A), (B) A–B dimer interface. (D), (E) A–C dimer interface. The A subunit is shown in surface representation painted with yellow and white for hydrophobic and the other residues, respectively. The B and C subunits are shown by Cα-trace colored with pink in (A) and (D), respectively. Residues involved in the dimer interaction are labeled in (B) and (E). (C), (F) The A–B (C) and A–C (F) dimers viewed from the opposite side of both C-termini (viewed from the top of the model in (A) and in (D), respectively). The hydrophobic (same color as (A)) and the electrostatic potential (red, negative; blue, positive) surfaces are shown in left and right panels, respectively. The Cα-trace of the dimer is in the middle panel. (TIF) [file pbio.2002281.s008.tif]

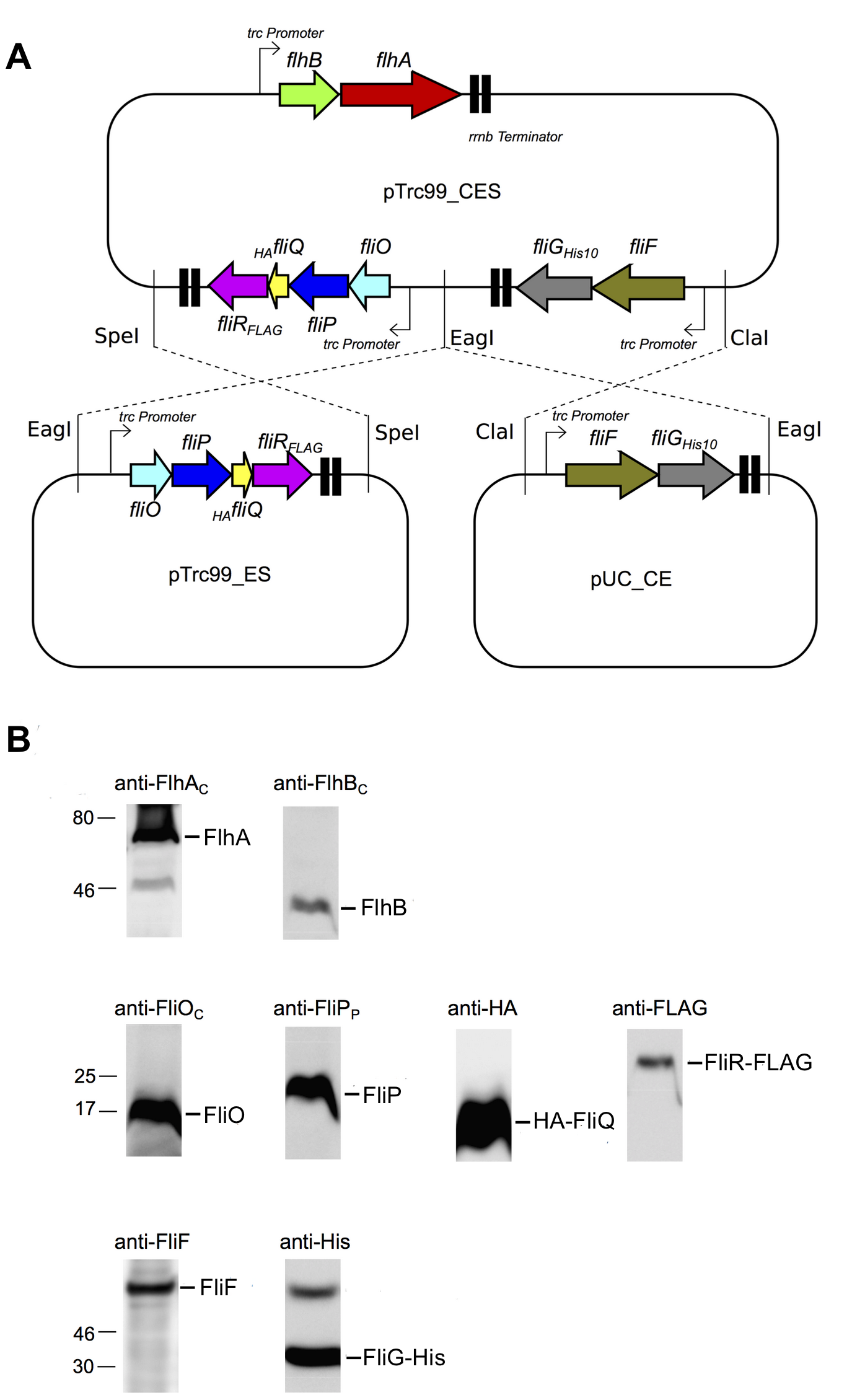

Supplement: S10 Fig — (A) Schematic diagram of plasmid construction of pKY079. (B) Expression of FlhA, FlhB, FliF, FliG-His, FliO, FliP, HA-FliQ and FliR-FLAG as judged by immunoblotting with anti-FlhAC, anti-FlhBC, anti-FliF, anti-His, anti-FliOC, anti-FliPP, anti-HA and anti-FLAG antibodies, respectively. (TIF) [file pbio.2002281.s010.tif]
