# Supplementary material for: Assembly and stoichiometry of the core structure of the bacterial flagellar type III export gate complex
Source: PLoS Biol. 2017 Aug 3;15(8):e2002281. doi: 10.1371/journal.pbio.2002281 (PMC5542437; doi:10.1371/journal.pbio.2002281)
Supplement: S1 Table — (DOCX) [file pbio.2002281.s011.docx]

**S1 Table. Strains and plasmids used in this study**

| Strains and Plasmids | Relevant characteristics | Source or reference |
| --- | --- | --- |
| *E. coli* |  |  |
| BL21 (DE3) | Host for overexpression from the T7 promotor | Novagen |
|  |  |  |
| *Salmonella* |  |  |
| EM2269 | *fliO22348::3xFLAG, fliP8004::3xHA* | M. Erhardt |
| SJW1368 | ∆(*cheW-flhD*) | 1 |
| TH10548 | ∆*fliO* | K. T. Hughes |
| TH10549 | ∆*fliP* | K. T. Hughes |
|  |  |  |
| Plasmid |  |  |
| pUC19 | Expression vector | Invitrogen |
| pBAD24 | Expression vector | 2 |
| pTrc99AFF4 | Expression vector | 3 |
| pTrcCES | Expression vector | This study |
| pKY045 | pET15b/ *Tm*-FliP(110-188) | 4 |
| pKY010 | pUC19/ HA-FliP | This study |
| pKY010(P115A) | pUC19/ HA-FliP(P115A) | This study |
| pKY010(E125A) | pUC19/ HA-FliP(E125A) | This study |
| pKY010(F137A) | pUC19/ HA-FliP(F137A) | This study |
| pKY010(F150A) | pUC19/ HA-FliP(F150A) | This study |
| pKY010(L170A) | pUC19/ HA-FliP(L170A) | This study |
| pKY010(P172A) | pUC19/ HA-FliP(P172A) | This study |
| pKY010(A173S) | pUC19/ HA-FliP(A173S) | This study |
| pKY010(S177A) | pUC19/ HA-FliP(S177A) | This study |
| pKY010(E178A) | pUC19/ HA-FliP(E178A) | This study |
| pKY010(L179A) | pUC19/ HA-FliP(L179A) | This study |
| pKY010(A182S) | pUC19/ HA-FliP(A182S) | This study |
| pKY010(F183A) | pUC19/ HA-FliP(F183A) | This study |
| pKY041 | pBAD24/ HA-FliP | This study |
| pKY041(P115A) | pBAD24/ HA-FliP(P115A) | This study |
| pKY041(E125A) | pBAD24/ HA-FliP(E125A) | This study |
| pKY041(F137A) | pBAD24/ HA-FliP(F137A) | This study |
| pKY041(F150A) | pBAD24/ HA-FliP(F150A) | This study |
| pKY041(L170A) | pBAD24/ HA-FliP(L170A) | This study |
| pKY041(P172A) | pBAD24/ HA-FliP(P172A) | This study |
| pKY041(A173S) | pBAD24/ HA-FliP(A173S) | This study |
| pKY041(S177A) | pBAD24/ HA-FliP(S177A) | This study |
| pKY041(E178A) | pBAD24/ HA-FliP(E178A) | This study |
| pKY041(L179A) | pBAD24/ HA-FliP(L179A) | This study |
| pKY041(A182S) | pBAD24/ HA-FliP(A182S) | This study |
| pKY041(F183A) | pBAD24/ HA-FliP(F183A) | This study |
| pKY069 | pTrc99AFF4/ His-FliP | This study |
| pKY070 | pTrc99AFF4/ FliO + His-FliP | This study |
| pKY071 | pTrc99AFF4/ FliO + His-FliP(F137A) | This study |
| pKY085 | pTrc99AFF4/ FliO + His-FliP(F150A) | This study |
| pKY082 | pTrc99AFF4/ FliO + His-FliP(E178A) | This study |
| pKY073 | pTrc99AFF4/ FliO | This study |
| pKY074 | pTrc99AFF4/ HA-FliP | This study |
| pKY086 | pTrc99AFF4/ FliO + His-FliP + HA-FliQ | This study |
| pKY087 | pTrc99AFF4/ FliO + His-FliP + FliR-FLAG | This study |
| pKY088 | pTrc99AFF4/ His-FliP + FliR-FLAG | This study |
| pKY089 | pTrc99AFF4/ His-FliP+ HA-FliQ + FliR-FLAG | This study |
| pKY077 | pTrc99AFF4/ FliO + His-FliP + HA-FliQ + FliR-FLAG | This study |
| pKY078 | pTrc99CES/ FlhB + FlhA + FliO + His-FliP + HA-FliQ + FliR-FLAG | This study |
| pKY079 | pTrc99CES/ FlhB + FlhA + FliO + FliP + HA-FliQ + FliR-FLAG + FliF + FliG-His | This study |
| pSup-pBpa | amber stop codon suppression plasmid | 5 |
| pSB3410 | pT10/ SpaP^84FLAG^ | 6 |
| pMIB6364 | pT10/ FliO + FliP^157FLAG^ + FliQ + FliR | This study |

| pMIB6376 | pT10/ FliO + FliP^157FLAG^_L51X_ + FliQ + FliR | This study |
| --- | --- | --- |
| pMIB6376 | pT10/ FliO + FliP^157FLAG^_L52X_ + FliQ + FliR | This study |

| pMIB6378 | pT10/ FliO + FliP^157FLAG^_M123X_ + FliQ + FliR | This study |
| --- | --- | --- |
| pMIB6379 | pT10/ FliO + FliP^157FLAG^_Q124X_ + FliQ + FliR | This study |
| pMIB6380 | pT10/ FliO + FliP^157FLAG^_L127X_ + FliQ + FliR | This study |
| pMIB6471 | pT10/ FliO + FliP^157FLAG^_F137X_ + FliQ + FliR | This study |
| pMIB6472 | pT10/ FliO + FliP^157FLAG^_F150X_ + FliQ + FliR | This study |
| pMIB6473 | pT10/ FliO + FliP^157FLAG^_R152X_ + FliQ + FliR | This study |
| pMIB6474 | pT10/ FliO + FliP^157FLAG^_A154X_ + FliQ + FliR | This study |
| pMIB6475 | pT10/ FliO + FliP^157FLAG^_S156X_ + FliQ + FliR | This study |
| pMIB6476 | pT10/ FliO + FliP^157FLAG^_P158X_ + FliQ + FliR | This study |
| pMIB6491 | pT10/ FliO + FliP^157FLAG^ | This study |

| pMIB6493 | pT10/ FliO + FliP^157FLAG^_T52X_ | This study |
| --- | --- | --- |

| pMIB6493 | pT10/ FliO + FliP^157FLAG^_F150X_ | This study |
| --- | --- | --- |
| pMIB6494 | pT10/ FliO + FliP^157FLAG^_R152X_ | This study |
| pMIB6495 | pT10/ FliO + FliP^157FLAG^_S156X_ | This study |
| pMIB6496 | pT10/ FliO + FliP^157FLAG^_P158X_ | This study |
| pMIB6498 | pT10/ FliO^FLAG^ + FliP^HA^ | This study |
| pMIB6541 | pT10/ FliO^FLAG^ + FliP^HA^_R152X_ | This study |

**References**

1. Ohnishi K, Ohto Y, Aizawa S, Macnab RM, Iino T. FlgD is a scaffolding protein needed for flagellar hook assembly in *Salmonella typhimurium*. J Bacteriol. 1994;176: 2272–2281. doi: 10.1128/jb.176.8.2272-2281.1994
2. Guzman LM, Belin D, Carson MJ, Beckwith J. Tight regulation, modulation, and high-level expression by vectors containing the arabinose pBAD promoter. J Bacteriol. 1995;177: 4121–4130. doi: 10.1128/jb.177.14.4121-4130.1995.
3. Ohnishi K, Fan F, Schoenhals GJ, Kihara M, Macnab RM. The FliO, FliP, FliQ, and FliR proteins of *Salmonella typhimurium*: putative components for flagellar assembly. J Bacteriol. 1997;179: 6092–6099. doi: 10.1128/jb.179.19.6092-6099.1997.
4. Fukumura T, Furukawa Y, Kawaguchi T, Saijo-Hamano Y, Namba K, Imada K, et al. Crystallization and preliminary X-ray analysis of the periplasmic domain of FliP, an integral membrane component of the bacterial flagellar type III protein export apparatus. Acta Crystallogr Sect F Struct Biol Cryst Commun. 2014;70: 1215–1218. doi: 10.1107/S2053230X14014678.
5. Ryu Y, Schultz PG. Efficient incorporation of unnatural amino acids into proteins in *Escherichia coli.* Nat Methods. 2006;3:263–265. doi: [10.1038/nmeth864](https://dx.doi.org/10.1038/nmeth864).
6. Zilkenat S, Franz-Wachtel M, Stierhof YD, Galán JE, Macek B, Wagner S. Determination of the stoichiometry of the complete bacterial type III secretion needle complex using a combined quantitative proteomic approach. Mol Cell Proteomics. 2016;15: 1598–1609. doi: 10.1074/mcp.M115.056598.
