# Supplementary material for: Assembly and stoichiometry of the core structure of the bacterial flagellar type III export gate complex
Source: PLoS Biol. 2017 Aug 3;15(8):e2002281. doi: 10.1371/journal.pbio.2002281 (PMC5542437; doi:10.1371/journal.pbio.2002281)
Supplement: S2 Table — (DOCX) [file pbio.2002281.s012.docx]

**Table 2. X-ray refinement statistics**

| Measurements | Value |
| --- | --- |
| Resolution (Å) | 42.9 - 2.4 |
| No. of reflections | 30084 |
| Rwork/Rfree | 0.211 / 0.258 |
| No. atoms |  |
| Protein | 4354 |
| Water | 397 |
| B-factor |  |
| Protein | 46.3 |
| Water | 54.8 |
| R.m.s deviations |  |
| Bond lengths (Å) | 0.004 |
| Bond angles (º) | 0.778 |

^#^*R*_merge_ = Σ**_h_** Σ*_l_* |*I***_h_***_l_*- <*I***_h_**>|/ Σ**_h_** Σ*_l_* <*I***_h_***>*, where *I_l_* is the *l*th observation of reflection **h** and <*I***_h_**> is the weighted average intensity for all observations *l* of reflection **h***.*

^+^*R*_ano_ = Σ**_h_** |<*I*(**h**+)> - <*I*(**h**-)*>*|/ Σ**_h_** (<*I*(**h**+)> + <*I*(**h**-)*>*), where <*I*(**h**+)> and <*I*(**h**-)> correspond to the average intensities of each Friedel pair for reflection **h**.

R_w_= ∑ ||F_o_| - |F_c_|| / ∑ |F_o_|, same as R_free_ but calculated on 5% of data set aside for refinement.
